# Alterations in zonal distribution and plasma membrane localization of hepatocyte bile acid transporters in patients with NAFLD

**DOI:** 10.1097/HC9.0000000000000377

**Published:** 2024-02-14

**Authors:** William A. Murphy, Anna Mae Diehl, Matthew Shane Loop, Dong Fu, Cynthia D. Guy, Manal F. Abdelmalek, Georgia Sofia Karachaliou, Noora Sjöstedt, Sibylle Neuhoff, Paavo Honkakoski, Kim L. R. Brouwer

**Affiliations:** 1 Division of Pharmacotherapy and Experimental Therapeutics, UNC Eshelman School of Pharmacy, University of North Carolina at Chapel Hill, Chapel Hill, North Carolina, USA; 2 Division of Gastroenterology and Hepatology, Duke University Medical Center, Durham, North Carolina, USA; 3 Department of Health Outcomes Research and Policy, Harrison College of Pharmacy, Auburn University, Auburn, Alabama, USA; 4 Department of Pathology, Duke University, Durham, North Carolina, USA; 5 Division of Gastroenterology and Hepatology, Mayo Clinic, Rochester, Minnesota, USA; 6 Division of Pharmaceutical Biosciences, Faculty of Pharmacy, University of Helsinki, Helsinki, Finland; 7 Simcyp Division, Certara UK Ltd., Sheffield, United Kingdom; 8 School of Pharmacy, Faculty of Health Sciences, University of Eastern Finland, Kuopio, Finland

## Abstract

**Background:**

NAFLD is highly prevalent with limited treatment options. Bile acids (BAs) increase in the systemic circulation and liver during NAFLD progression. Changes in plasma membrane localization and zonal distribution of BA transporters can influence transport function and BA homeostasis. However, a thorough characterization of how NAFLD influences these factors is currently lacking. This study aimed to evaluate the impact of NAFLD and the accompanying histologic features on the functional capacity of key hepatocyte BA transporters across zonal regions in human liver biopsies.

**Methods:**

A novel machine learning image classification approach was used to quantify relative zonal abundance and plasma membrane localization of BA transporters (bile salt export pump [BSEP], sodium-taurocholate cotransporting polypeptide, organic anion transporting polypeptide [OATP] 1B1 and OATP1B3) in non-diseased (n = 10), NAFL (n = 9), and NASH (n = 11) liver biopsies. Based on these data, membrane-localized zonal abundance (MZA) measures were developed to estimate transporter functional capacity.

**Results:**

NAFLD diagnosis and histologic scoring were associated with changes in transporter membrane localization and zonation. Increased periportal BSEP_MZA_ (mean proportional difference compared to non-diseased liver of 0.090) and decreased pericentral BSEP_MZA_ (−0.065) were observed with NASH and also in biopsies with higher histologic scores. Compared to Non-diseased Liver, periportal OATP1B3_MZA_ was increased in NAFL (0.041) and NASH (0.047). Grade 2 steatosis (mean proportional difference of 0.043 when compared to grade 0) and grade 1 lobular inflammation (0.043) were associated with increased periportal OATP1B3_MZA_.

**Conclusions:**

These findings provide novel mechanistic insight into specific transporter alterations that impact BA homeostasis in NAFLD. Changes in BSEP_MZA_ likely contribute to altered BA disposition and pericentral microcholestasis previously reported in some patients with NAFLD. BSEP_MZA_ assessment could inform future development and optimization of NASH-related pharmacotherapies.

## INTRODUCTION

NAFLD is an emerging global health crisis with an estimated adult prevalence of 30% and is rapidly becoming the leading cause of liver transplantation in the United States.^1,2^ NAFLD encompasses a broad clinical spectrum ranging from NAFL to NASH. NASH can progress to HCC and cirrhosis, both of which are associated with high rates of morbidity and mortality.^1,2^ Importantly, regulatory-approved pharmacologic treatments for NAFLD are still lacking; this is likely due to the pathophysiological complexity of NAFLD coupled with our incomplete understanding of its development and progression.^3,4^

Bile acids (BAs) are synthesized in the liver, facilitate intestinal lipid absorption, and act as signaling molecules to regulate energy, glucose, and immune function. Notably, altered BA homeostasis has been proposed to play a central role in NAFLD pathophysiology and progression.^3,5,6^ Hydrophobic BAs, which can cause liver injury,^7,8^ are increased in the systemic circulation, hepatic tissue, and feces of patients with NASH.^9–14^ Although several drugs targeting the BA-activated nuclear receptor farnesoid X receptor (FXR/*NR1H4*) are currently in clinical development to treat NAFLD,^15^ the mechanistic causes of altered BA homeostasis during NAFLD development and progression remain largely unknown.^5^

Transporters are membrane-bound proteins that facilitate substrate movement across plasma membranes and play a key role in maintaining the homeostasis of endogenous compounds such as BAs. BA homeostasis is tightly regulated, in part via enterohepatic recycling, which involves several enterocyte and hepatocyte transporters working in concert to recycle ~95% of all secreted BAs. The principal BA transporters in the human hepatocyte are the basolateral sodium-taurocholate cotransporting polypeptide polypeptide (NTCP/*SLC10A1*; basolateral uptake) and the apical bile salt export pump (BSEP/*ABCB11*; canalicular excretion). BSEP is the rate-limiting step for hepatocyte BA secretion into the bile canaliculi.^16^ BSEP dysfunction due to genetic mutations or inhibition by endogenous metabolites or drugs can result in hepatotoxic cholestasis.^16^ NAFLD severity has been associated with reduced BSEP mRNA expression^17^ although no changes in BSEP protein abundance measured by mass spectrometry-based quantitative targeted absolute proteomics were observed in NAFLD.^18^ In humans, NTCP is the primary hepatocyte BA uptake transporter^19^ and the organic anion–transporting polypeptides (OATPs) contribute to the basolateral uptake of glycine-conjugated and taurine-conjugated BAs.^20,21^ NTCP mRNA and protein are downregulated during cholestasis,^16,22^ and NTCP and OATP1B protein are decreased during NAFLD progression.^18^

Hepatocyte transporters also are important in the disposition of many drugs and may be the rate-determining step in the elimination of medications taken by patients with NAFLD for comorbidities (e.g., statins for hyperlipidemia).^23^ Impaired transport function due to disease can result in systemic and/or tissue accumulation of drugs or endogenous compounds.^24^ Changes in hepatocyte transporter function in patients with NAFLD may impact the concentration-time profiles of some medications and result in adverse drug reactions.^23^ For example, BSEP inhibition is associated with BA-mediated DILI^25^ and patients with NAFLD are suggested to have increased susceptibility to DILI.^7^ Several mechanisms have been proposed to support this hypothesis including reduced mitochondrial function in NAFLD.^26^ In theory, this would impair BSEP-mediated hepatocyte BA efflux, which is an ATP-dependent process.^7^ Therefore, it is necessary to have a comprehensive understanding of factors that may impact hepatocyte transporter function in patients with NAFLD.

Hepatocyte transporter protein abundance is commonly used in physiologically based pharmacokinetic/pharmacodynamic models to predict drug disposition and pharmacologic/toxicologic responses. Notable changes in transporter abundance have been documented in NAFLD.^27^ Protein abundance tends to correlate better with transport function than mRNA levels.^27^ A common assumption is that all transport protein measured within a sample is localized to the hepatocyte plasma membrane and equally distributed across zonal regions of the liver. However, immunohistochemistry studies have revealed that hepatocyte transporters are not always entirely localized on the hepatocyte membrane,^22,28^ particularly in NAFLD.^29,30^ Immunohistochemistry data are qualitative in nature and thus limited in their application.^23^ This is an important knowledge gap as transporters must be properly inserted into the plasma membrane to facilitate efficient substrate uptake or efflux.^27^ Furthermore, the abundance of hepatocyte transporters is not necessarily homogenous across the liver acinus under nonpathologic conditions.^22,31,32^

Liver zonation is an important aspect of NAFLD and disease progression.^33,34^ It may also explain variability in clinical presentation and pharmacologic outcomes in this population.^35^ For example, the NAFLD drug target FXR is thought to exhibit preferential activity in the pericentral region.^35,36^ This notion aligns with the findings of the Farnesoid X nuclear receptor ligand obeticholic acid for non-cirrhotic, non-alcoholic steatohepatitis (FLINT) clinical trial, which demonstrated that the administration of obeticholic acid, an FXR agonist, specifically improved liver inflammation associated with NASH in the lobular region (zones 2–3), but did not significantly affect inflammation in the portal area (zone 1).^37^ While changes in the zonal abundance patterns of hepatocyte transporters have yet to be evaluated in NAFLD, proper characterization of transporter zonation could have important implications for improving pharmacokinetic and pharmacodynamic or toxicodynamic predictions in this population.^38^

The primary aim of this study was to quantitatively assess the zonal abundance (tissue level) and plasma membrane localization (cellular level) of 4 key hepatocyte BA transporters (BSEP, NTCP, OATP1B1, and OATP1B3) in non-diseased livers (NDLs) and livers at various stages of NAFLD progression. To achieve this aim, we developed a methodological framework that incorporates three-dimensional (3D) fluorescence immunohistochemical imaging with machine learning image classification and analysis. Quantifiable alterations in the plasma membrane localization, zonal abundance, and membrane-localized zonal abundances (MZAs) of select transporters were discovered in patients with NAFLD and associated with histologic scoring. Our findings provide valuable mechanistic insight into altered BA homeostasis in NAFLD. Furthermore, these data may be used to optimize medication dosing/patient stratification in the population with NAFLD during drug development and in clinical practice.

## METHODS

A detailed description of the methodology is provided in the Supplemental Methods, http://links.lww.com/HC9/A781. Written informed consent was provided by all subjects and the study protocol conformed to the 2013 Declaration of Helsinki and 2018 Declaration of Istanbul ethical guidelines. Ethical approval for this study was obtained from the Duke University Health System Institutional Review Board (Protocol # 00005368).

### Study population

The overall study cohort composed of 10 subjects with normal or nonpathogenic liver histology after being evaluated for suspected liver disease (defined as NDL), 9 subjects with biopsy-proven NAFL, and 11 subjects with biopsy-proven NASH without cirrhosis. Liver histologic assessments were performed by board-certified clinical hepatopathologists at Duke University Hospital in accordance with the NASH Clinical Research Network classification system.^39^ Hepatic fibrosis stages 1a, 1b, and 1c were grouped together. Histologic-based diagnoses were defined as follows: NDL as liver biopsies with NAFLD activity scores of 0; NAFL as steatosis ≥5% and absence of necroinflammation, ballooned hepatocytes, or fibrosis; and NASH as steatosis ≥5% with the presence of lobular inflammation with hepatocellular ballooning and/or fibrosis.^40^ Summary demographic and histology data for the study population are provided in Table 1. As expected, some individuals had multiple diagnosed metabolic syndrome–associated comorbidities. In total, 120 glass slides mounted with 10-μm sectioned formalin-fixed paraffin-embedded percutaneous liver needle biopsies (samples) originating from the 30 study subjects were acquired (4 matching samples per subject). Investigators performing confocal imaging of the samples and data acquisition were blinded to each subject’s NAFLD status and demographic/histologic data. Unique numerical patient identification codes (sample IDs) were made available to ensure all four transporters were assessed for each biopsy. Investigators were unblinded prior to data cleaning and analysis.

**TABLE 1:** Demographic and histologic patient characteristics

Patient characteristics	Non-Diseased Liver^a^ (n = 10)	NAFL^a^ (n = 9)	NASH^a^ (n = 11)
Age, y, median (IQR) [range]^b^	41 (38.3–48.3) [35–65]	57 (53–59) [32–65]	50 (39–54.5) [21–64]
Female sex, n (%)^b^	5 (50)	4 (44.4)	6 (54.5)
Body mass index, kg/m^2^, median (IQR) [range]^b^	39.6 (30.6–48.9) [21.1–53.9]	30.9 (27.8–33.6) [25.7–38.8]	36.9 (28.7–38.7) [23.5–46.9]
Self-reported race, n (%)			
White	4 (40)	9 (100)	8 (72.7)
Black	4 (40)	0 (0)	2 (18.2)
Asian	1 (10)	0 (0)	1 (9.1)
Multiracial	1 (10)	0 (0)	0 (0)
Medical history at initial visit, n (%)^c^			
Diabetes mellitus	4 (40)	2 (22.2)	4 (40^d^)
Hyperlipidemia	4 (44.4^d^)	6 (66.7)	6 (60^d^)
Hypertension	3 (30)	5 (55.6)	8 (80^d^)
Histological assessment of liver biopsy^e^, n			
Steatosis grade, (0/1/2/3)^f^	10/0/0/0	0/2/5/2	0/5/3/3
Lobular inflammation grade, (0/1/2/3)	10/0/0/0	0/8/1/0	0/9/2/0
Hepatocellular ballooning grade, (0/1/2)	10/0/0	9/0/0	1/7/3
NAFLD activity score (NAS), (0/1/2/3/4/5/6)	10/0/0/0/0/0/0	0/0/2/5/1/1/0	0/0/0/3/4/3/1
Fibrosis stage, (0/1/2/3/4)	10/0/0/0/0	9/0/0/0/0	6/2/3/0/0
Fibrosis stage >0^g^, n (%)	0 (0)	0 (0)	5 (45.5)

a Diagnosis performed in accordance with the NASH Clinical Research Network classification system: non-diseased liver = NAFLD activity score of 0; NAFL = steatosis > 5% (S1–S3) without hepatocellular ballooning or fibrosis; NASH = steatosis >5% with lobular inflammation and hepatocellular ballooning and/or fibrosis.

b No statistically significant differences (*p*<0.05) across histologic-based NAFLD diagnostic groups.

c Some individuals across all 3 histologic-based diagnosis groups had more than 1 diagnosed metabolic syndrome–associated comorbidity resulting in % sum values >100%.

d One subject with missing data (resulting in n = 9 for NDL and n = 10 for NASH when assessing diagnostic % of these comorbidities).

e NASH Clinical Research Network histologic scoring system used for biopsy assessment.

f Steatosis grade equates to observed % steatosis on biopsy as follows:

(0/1/2/3) = (<5%/5%–33%/34%–66%/>66%).

g Fibrosis was assessed as a dichotomous variable (F0/F1-F2) due to the small sample size and limited statistical power per stage.

Abbreviation: NAS, NAFLD activity score.

### Immunofluorescence and image acquisition

All primary and secondary antibodies used for immunofluorescence staining of formalin-fixed paraffin-embedded liver biopsies are listed in Table 2. Individual samples were stained separately for each transporter of interest (BSEP, NTCP, OATP1B1, OATP1B3). Isotype-matched control antibodies (Supplemental Table S1, http://links.lww.com/HC9/A781) were used as negative controls. Image acquisition was performed with Zeiss Zen software (version 2.3 SP1) using a Zeiss LSM 880 confocal laser scanning microscope (Carl Zeiss AG, Oberkochen, Germany) and an EC Plan-Neofluar ×40/1.30 Oil DIC M27 objective. Images were acquired using 405-nm (DAPI), 561-nm (Alexa Fluor 568), and 633-nm (Alexa Fluor 647) laser lines. Three randomly selected areas that included both portal tract (PT) and central vein (CV) structures were imaged within each sample, except in one NDL biopsy where only 2 viable areas were available. PT and CV structures were subjectively identified using the DAPI nuclei stain. Tile scanning was performed using various *x* and *y* dimensions to adequately capture individual PT-to-CV axes with a z-stack interval of 0.55 μm to generate 3D images. At least 18 z-stack slices (~9.9 μm) were acquired for each image to capture all desired fluorescence signals. Further details on immunofluorescence staining and image acquisition are included in Supplemental Methods, http://links.lww.com/HC9/A781.

**TABLE 2:** Description of primary and secondary antibodies used in this study

Antibody (host species-target)	Catalog number	Supplier	Dilution	Antibody type/target class
Mouse-anti-BSEP	sc-74500	Santa Cruz Biotechnology (Dallas, TX)	1:40	Primary/transporter
Rabbit-anti-NTCP	GTX17693	GeneTex (Irvine, CA)	1:30	Primary/transporter
Mouse-anti-OATP1B1	NB10074481	Novus Biologicals (Littleton, CO)	1:100	Primary/transporter
Rabbit-anti-OATP1B3	Custom-made	Dr. Wei Yue (College of Pharmacy, The University of Oklahoma Health Sciences Center, Oklahoma City, Oklahoma, USA)	1:250	Primary/transporter
Rabbit-anti-Na^+^/K^+^ ATPase	ab185065	Abcam (Waltham, MA)	1:500	Primary/membrane marker
Mouse-anti-Na^+^/K^+^ ATPase	sc-48345	Santa Cruz Biotechnology	1:500	Primary/membrane marker
Rabbit-anti-CD13	ab108382	Abcam	1:750	Primary/membrane marker
Goat-anti-rabbit IgG Alexa Fluor 568	A11036	Invitrogen (Waltham, MA)	1:100	Secondary
Goat-anti-mouse IgG Alexa Fluor 647 (not cross-adsorbed against mouse IgM)	A21235	Invitrogen	1:100	Secondary

Abbreviations: BSEP, bile salt export pump; CD13, aminopeptidase N; NTCP, sodium-taurocholate cotransporting polypeptide; OATP, organic anion transporting polypeptide.

### Image analysis–Transporter zonal abundance and plasma membrane localization quantification

Transporter 3D surfaces in Imaris were transformed to mask their respective source image fluorescence channel (“masked fluorescence images”) and then exported into Fiji (ImageJ v. 1.53t). 3D fluorescence intensity data were transformed into *z*-axis sum fluorescence projections. Mean fluorescence intensity throughout the y-axis at each pixel width (0.208 μm) was calculated across the *x*-axis from PT to CV. Within each image, fluorescence intensity data were pooled into 3 equidistant hepatic regions (zones 1–3) across the PT-to-CV axis.^41^ Summed fluorescence intensity values within each region were divided by the total sum fluorescence intensity of the image to obtain relative zonal abundance measures (range 0–1). Carl Zeiss image files were uploaded into Imaris Image Analysis software (v. 9.9.1, Bitplane, Zurich, Switzerland). PT and CV structures were aligned along the *x*-axis by rotating the image before defining a rectangular region of interest (ROI) within the constructs of the software. The *x*-axis dimension (length) for each ROI spanned from the midpoint of the CV to the edge of the connective tissue surrounding the PT (beginning of portal parenchyma) (Supplemental Figure S1A, http://links.lww.com/HC9/A781). The *y*-axis dimension (width) was defined as 3 times the mean CV diameter as measured across both the *x*-axis and *y*-axis because the CV was rarely a symmetrical circle. 3D surface rendering and subsequent machine learning image classification of transporter and plasma membrane marker fluorescence signal were performed using Imaris and Fiji Labkit (ImageJ version 1.53t). The primary purpose of machine learning image classification was to exclude background and nonhepatocyte signal (e.g., sinusoidal cavity) for the transporter marker, and nonplasma membranous staining (e.g., intracellular) for the plasma membrane marker (Supplemental Figure S2, http://links.lww.com/HC9/A781). At least 3 iterations of training data using manual surface classification were input for each sample. Images that could not be adequately segmented by machine learning were manually segmented in Imaris. Specific parameters used for surface rendering in Imaris are provided in Supplemental Table S2, http://links.lww.com/HC9/A781. Percent (%) volume overlap of the transporter 3D surface with the plasma membrane marker 3D surface was determined with Imaris object-object statistics as follows: total transporter surface volume directly overlapping with the plasma membrane marker surface (within 208 nm for Na^+^/K^+^-ATPase; within 400 nm for aminopeptidase N [CD13]) was divided by the total transporter surface volume within the entire ROI and then multiplied by 100 to obtain a percentage (0%–100%); these data were defined as overall (nonzonally segmented) membrane localization. Transporter surface volume not overlapping with the membrane marker was assumed to be localized to intracellular vesicles or compartments.^27^ To obtain zonal transporter plasma membrane localization data, the mean membrane localization value was determined for 3 equidistant regions (zones 1–3) along the *x*-axis within a defined ROI. Additional detail on image analysis is provided in Supplemental Methods, http://links.lww.com/HC9/A781.

### MZA quantification

Mean zonal transporter membrane localization measurements obtained from zones 1–3 were used to adjust zonal transporter abundance data. Summed fluorescence intensity values from each hepatic zone were multiplied by the mean membrane localization percentage (%) for that corresponding zone and image to obtain MZA measures for each transporter. Adjusted abundance measures within each zone were divided by the total adjusted abundance amount to obtain relative transporter MZA measures (ranging from 0 to 1).

### Data analysis

Plasma membrane localization data from Imaris and zonal fluorescence intensity data from Fiji were saved in .csv format and then uploaded into RStudio (Version 2022.12.0+353; R version 4.3) for data cleaning, statistical analysis, data visualization, and Bayesian regression modeling. Bayesian modeling was performed using the *brms* package (v. 2.18.0).

To assess the specific impact of histologic-based NAFLD diagnosis and histologic features on each outcome of interest after adjusting for individual demographic characteristics, Bayesian generalized linear mixed modeling (B-GLMM) was performed. A Bayesian approach was chosen over more traditional statistical methods to provide improved computational performance and stability for fitting complex multilevel models.^42,43^ Statistical testing was not performed on the raw data. All available measurements for each sample were used for Bayesian regression analysis. Relevant demographic covariates (i.e., age, sex, race, and body mass index) were included as fixed effects. A random effect term was included for sample ID to account for interindividual variability. To align with distributional assumptions, all models assessing plasma membrane localization (0%–100%) as the outcome variable were specified using a Beta family distribution, while those assessing zonal abundance and MZA (0–1; values across all 3 zones are constrained to a total sum of 1) as the outcome variable used a Dirichlet family distribution. All estimated mean adjusted effect sizes for NAFLD diagnosis and histologic score are for a hypothetical subject of our median sample age (50 years) and body mass index (33.56 kg/m^2^) averaged over all available levels of race and sex. The median point estimate and 95% credible interval of the posterior distribution are presented throughout. Bayesian regression priors and model estimated adjusted mean differences are listed in Supplemental Tables S3, http://links.lww.com/HC9/A781 and S4–S6, http://links.lww.com/HC9/A781, respectively. Additional detail on Bayesian regression methodology is provided in Supplemental Methods, http://links.lww.com/HC9/A781.

All data and the corresponding R code files used for data cleaning, analysis, and Bayesian modeling are deposited on Zenodo (https://doi.org/10.5281/zenodo.10558835).

## RESULTS

### BSEP and OATP1B3 zonal abundance patterns changed with NAFLD diagnosis

**FIGURE 1:**
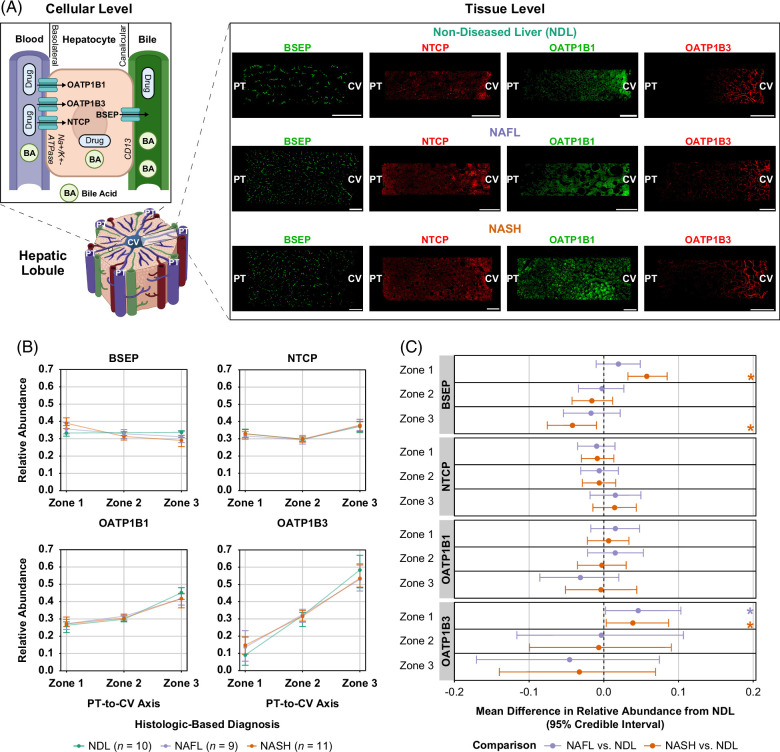
Relative zonal abundance patterns of BSEP and OATP1B3 are altered in NASH and NAFLD, respectively. (A) “Cellular Level” and “Hepatic Lobule” schematics created with BioRender.com depict hepatocyte transporters of interest on the basolateral (NTCP, OATP1B1, and OATP1B3) and canalicular (BSEP) plasma membrane domains. Na^+^/K^+^-ATPase (basolateral) and aminopeptidase N (CD13; canalicular) serve as domain-specific markers. “Tissue level” shows representative masked confocal fluorescence images of transporter zonal abundance. Scale bars, 100 μm. (B) Median (quartile 1; quartile 3) relative transporter abundance patterns along the PT-to-CV axis. Plotted are triplicate measures (where available) taken from different areas of each liver biopsy. (C) Mean marginal effect on relative zonal transporter abundances for NAFL and NASH biopsies compared to NDL. Median and 95% credible intervals of each posterior distribution are shown. *95% credible interval does not cross 0. Abbreviations: BA, bile acid; BSEP, bile salt export pump; CV, central vein; NDL, non-diseased liver; NTCP, sodium-taurocholate cotransporting polypeptide; OATP, organic anion transporting polypeptide; PT, portal tract.

To investigate whether the zonal expression patterns of transporters changed in NAFLD, tissue-level zonation data were obtained for BSEP, NTCP, OATP1B1, and OATP1B3 (Figure 1A). OATP1B3 was highly abundant in the pericentral region in NDL; OATP1B1 had less preferential pericentral expression than OATP1B3 (Figure 1B). BSEP and NTCP did not exhibit strong zonation in NDL biopsies (Figure 1B). Dirichlet B-GLMMs were applied to quantitatively estimate the specific impact of NAFLD on relative zonal transporter abundance. After adjusting for available demographic variables and interindividual variability across measurements, negligible differences in zonal abundance patterns of NTCP and OATP1B1 were observed in NAFL and NASH biopsies compared to NDL (Figure 1C). However, relative BSEP zonal abundance was increased in the periportal region (zone 1) and decreased in the pericentral region (zone 3) in NASH. Estimated proportional BSEP abundances (range 0–1) in zones 1 and 3 were 0.34 and 0.32 for NDL, respectively, and 0.40 and 0.28 for NASH biopsies, respectively. The absolute estimated differences of relative zone 1 and zone 3 BSEP abundance for NASH compared to NDL biopsies were 0.058 (median point estimate) and −0.042, respectively (Figure 1C). OATP1B3 was also increased in the periportal region in NAFLD; the estimated mean differences of relative zone 1 OATP1B3 abundance in NAFL and NASH compared to NDL were 0.046 and 0.039, respectively (Figure 1C).

### Alterations in zonal abundance patterns of BSEP, NTCP, and OATP1B3 were associated with histologic scoring

**FIGURE 2:**
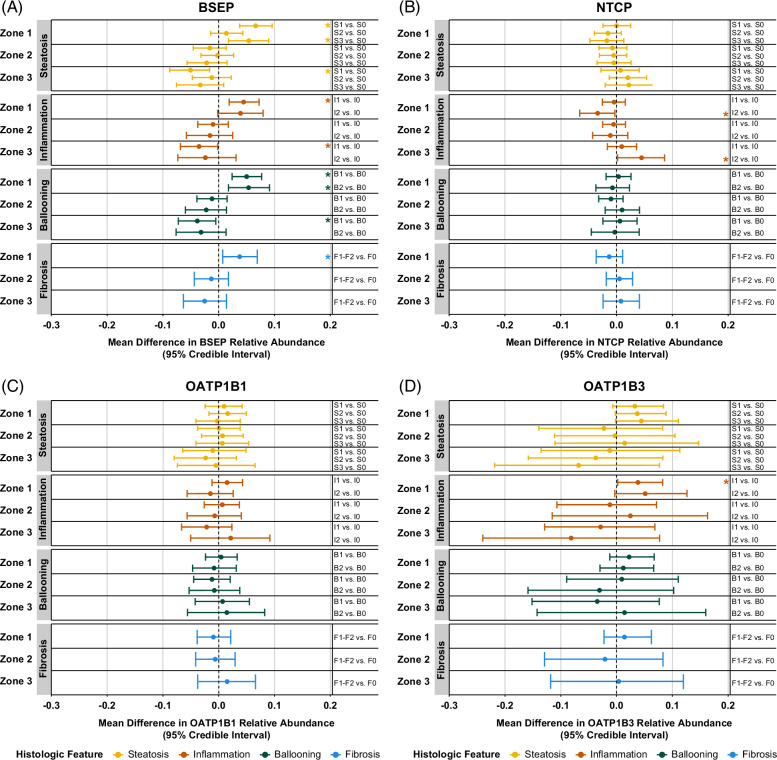
Differences in zonal abundance patterns of BSEP are associated with all histologic features of NAFLD, while zonal abundance patterns of NTCP and OATP1B3 are influenced by lobular inflammation. Mean marginal effects on relative zonal abundances of (A) BSEP, (B) NTCP, (C) OATP1B1, and (D) OATP1B3 were calculated by comparing biopsies of various histologic scores to a score of 0 with the corresponding histologic category. Median and 95% credible intervals of each posterior distribution are shown. The sample sizes for each histologic score are as follows: S0 (*n* = 10), S1 (*n* = 7), S2 (*n* = 8), S3 (*n* = 5); I0 (*n* = 10), I1 (*n* = 17), I2 (*n* = 3); B0 (*n* = 20), B1 (*n* = 7), B2 (*n* = 3); F0 (*n* = 25), F1-F2 (*n* = 5). *95% credible interval does not cross 0. Abbreviations: BSEP, bile salt export pump; NTCP, sodium-taurocholate cotransporting polypeptide; OATP, organic anion transporting polypeptide.

Given the variability in histologic features among patients within the same NAFLD diagnosis group (Table 1), we analyzed the specific influence of histologic scoring on transporter zonal abundance. Histologic grades and stages were abbreviated to indicate their corresponding histologic feature as follows: steatosis (S0, S1, S2, S3), lobular inflammation (I0, I1, I2), hepatocellular ballooning (B0, B1, B2), and fibrosis (F0, F1, F2). Dirichlet B-GLMM output for histologic grade for steatosis, lobular inflammation, hepatocellular ballooning, and fibrosis stage indicated an association of these features with increased periportal BSEP (Figure 2A). Estimated mean differences of S1, S3, I1, B1, B2, and F1-F2 biopsies for periportal BSEP abundance when compared to a score of 0 with the corresponding histologic category were 0.066, 0.054, 0.045, 0.050, 0.054, and 0.038, respectively. Notable decreases in pericentral BSEP abundance were also observed in S1, I1, and B1 biopsies with estimated mean differences when compared to a score of 0 with the corresponding histologic category of −0.050, −0.035, and −0.038, respectively.

Relative periportal and pericentral NTCP abundances were decreased and increased, respectively, in I2 biopsies (Figure 2B). Estimated mean differences for biopsies with an I2 score compared to I0 were −0.034 for zone 1 and 0.045 for zone 3. Negligible changes in relative OATP1B1 zonal abundance patterns were observed across histologic scores (Figure 2C). Periportal OATP1B3 zonal abundance was increased in I1 biopsies with an estimated mean difference compared to I0 biopsies of 0.039 (Figure 2D).

### Pericentral BSEP and periportal OATP1B3 plasma membrane localization were altered in NASH

**FIGURE 3:**
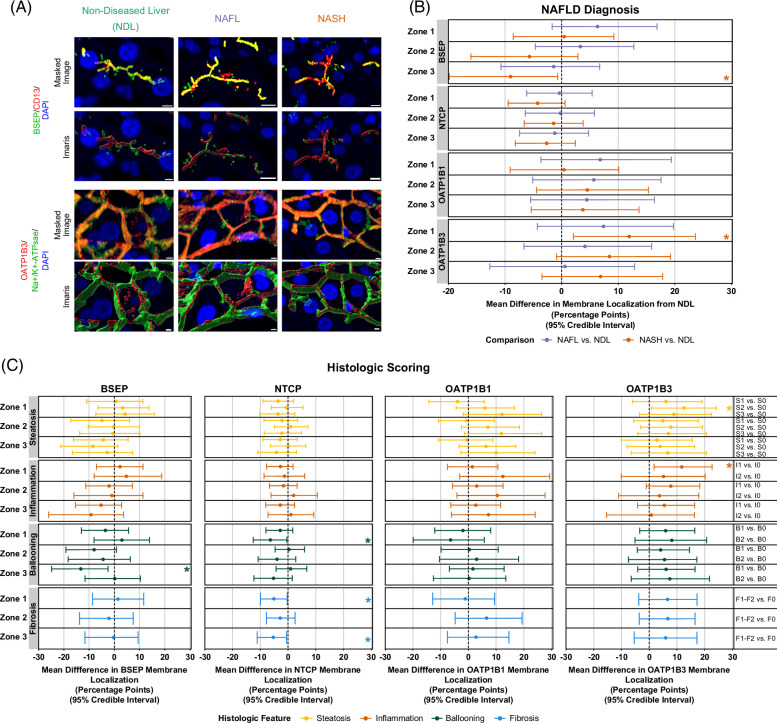
Plasma membrane localization of BSEP and OATP1B3 were altered in Zones 3 and 1, respectively, in NASH, while histologic features of hepatocyte injury (i.e., ballooning, fibrosis) are associated with zonal alterations in the plasma membrane localization of BSEP and NTCP; OATP1B3 Zone 1 membrane localization is influenced by steatosis and lobular inflammation. (A) Representative masked fluorescence images and corresponding three-dimensional volumetric surfaces of BSEP and OATP1B3 and plasma membrane markers derived using Imaris Image Analysis software. Yellow/orange coloration in masked fluorescence images indicates colocalization. Imaris software was used to quantify the volumetric overlap of surfaces. Scale bars, 10 μm. (B) The estimated mean difference of relative transporter membrane localization across zones 1–3 for NAFL and NASH biopsies compared to NDL and (C) various histologic scored biopsies compared to a score of 0 for the corresponding histologic category. (B–C) Median and 95% credible interval of each posterior distribution are shown. The sample sizes for each NAFLD diagnosis group are as follows: NDL (n = 10), NAFL (n = 9), and NASH (n = 11). Sample sizes for each histologic score are the same as detailed in the legend of Figure 2. *95% credible interval does not cross 0. Abbreviations: BSEP, bile salt export pump; NDL, non-diseased liver; NTCP, sodium-taurocholate cotransporting polypeptide; OATP, organic anion transporting polypeptide.

Next, we assessed the effect of NAFLD on the plasma membrane localization of hepatocyte transporters overall and in different zones. Imaris machine learning image classification was used to obtain volumetric transporter and plasma membrane marker surfaces from fluorescence imaging data (Figure 3A). No notable differences were observed in overall transporter plasma membrane localization in NAFL or NASH (Supplemental Figure S3A, http://links.lww.com/HC9/A781). A modest increase in overall OATP1B3 plasma membrane localization was observed in S2 compared to S0 biopsies (Supplemental Figure S3B, http://links.lww.com/HC9/A781). When assessing plasma membrane localization across hepatic zones, NASH biopsies had decreased pericentral BSEP and increased periportal OATP1B3 membrane localization (Figure 3B); the estimated mean differences expressed as percentage points were −9.0 and 12.0, respectively.

### Changes in BSEP, NTCP, and OATP1B3 zonal plasma membrane localization were associated with histologic features of liver injury

Histologic features indicative of liver injury were associated with alterations in BSEP and NTCP membrane localization across zones (Figure 3C). Specifically, hepatocellular ballooning was associated with decreased pericentral BSEP and decreased periportal NTCP membrane localization. The estimated mean difference expressed as a percentage point of B1 compared to a B0 score on Zone 3 BSEP membrane localization was −13.3, while that of a B2 score on zone 1 NTCP membrane localization was −6.4. Furthermore, periportal and pericentral NTCP membrane localization was decreased in subjects with NASH fibrosis. Estimated mean differences expressed as a percentage point of fibrosis compared to no fibrosis for zones 1 and 3 NTCP membrane localization were −5.1 and −5.3, respectively. Histologic scoring had a negligible impact on zonal OATP1B1 plasma membrane localization (Figure 3C). OATP1B3 plasma membrane localization was increased in Zone 1 of S2 and I1 biopsies with estimated mean differences expressed as a percentage point of 12.6 and 11.7 compared to S0 and I0 biopsies, respectively (Figure 3C).

### Periportal and pericentral BSEP membrane–localized zonal abundance (BSEP_MZA_) was altered in NASH and associated with higher histologic scores

**FIGURE 4:**
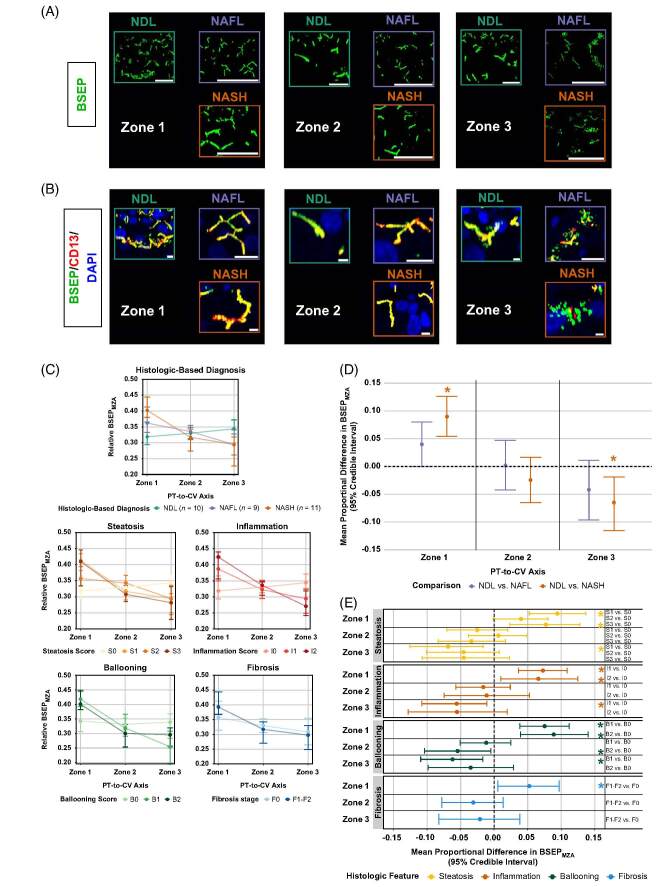
BSEP_MZA_ is altered in zones 1 and 3 in NASH; increased zone 1 BSEP_MZA_ is associated with the four main histologic features of NAFLD. (A, B) Representative three-dimensional images of BSEP fluorescence surface masks used to assess (A) zonal abundance (scale bars, 50 μm) and (B) membrane localization. Yellow/orange coloration in masked fluorescence images indicates colocalization. Imaris software was used to quantify the volumetric overlap of surfaces. Scale bars, 20 μm (C) Median (quartile 1; quartile 3) relative BSEP_MZA_ along the PT-to-CV) axis grouped by NAFLD diagnosis or histologic score. Plotted are triplicate measures (where available) taken from different areas of each liver biopsy. Sample sizes for each histologic score are the same as detailed in the legend of Figure 2. (D, E) Mean differences in zonal BSEP_MZA_ for (D) NAFL and NASH compared to NDL biopsies and (E) various histologic scored biopsies compared to a score of 0 for the corresponding histologic category. Median and 95% credible intervals of each posterior distribution are shown. *95% credible interval does not cross 0. Abbreviations: BSEP, bile salt export pump; CV, central vein; MZA, membrane-localized zonal abundance; NDL, non-diseased liver; PT, portal tract.

Based on our evaluation of how NAFLD and its histologic characteristics affect the abundance of transporters across hepatic zones and their localization on the plasma membrane, we developed a parameter that encompassed both features. This enabled us to quantitatively assess collective changes and better estimate potential alterations in transporter functional capacity across hepatic zones during NAFLD progression. To accomplish this, MZA estimates were developed using BSEP relative abundance (Figure 4A and Supplemental Figure S4A, http://links.lww.com/HC9/A781) and canalicular membrane localization (Figure 4B and Supplemental Figure S4B, http://links.lww.com/HC9/A781) measures obtained across hepatic zones 1–3 for all available liver biopsies. These data (Figure 4C) were used for Dirichlet B-GLMMs. Periportal increases and pericentral decreases in BSEP_MZA_ were observed in NASH biopsies; mean differences compared to NDL biopsies were 0.090 and −0.065, respectively (Figure 4D). NAFLD diagnosis had a negligible impact on zone 2 BSEP_MZA_.

Periportal BSEP_MZA_ was increased in biopsies of the highest reported scores across all NAFLD-related histologic features (Figure 4E). The estimated mean differences of S3, I2, B2, and F1-F2 biopsies for zone 1 BSEP_MZA_ when compared to a score of 0 with the corresponding histologic category were 0.077, 0.066, 0.089, and 0.052, respectively. Decreases in pericentral BSEP_MZA_ also were observed in S1, I1, and B1 biopsies; the estimated mean differences compared to S0, I0, and B0 were −0.069, −0.056, and −0.062, respectively. The only change observed with Zone 2 BSEP_MZA_ was a decrease in B2 biopsies with an estimated mean difference of −0.055 compared to B0 biopsies.

### Periportal OATP1B3 membrane–localized zonal abundance (OATP1B3_MZA_) was increased in NAFL and NASH with changes predominantly influenced by steatosis and lobular inflammation

**FIGURE 5:**
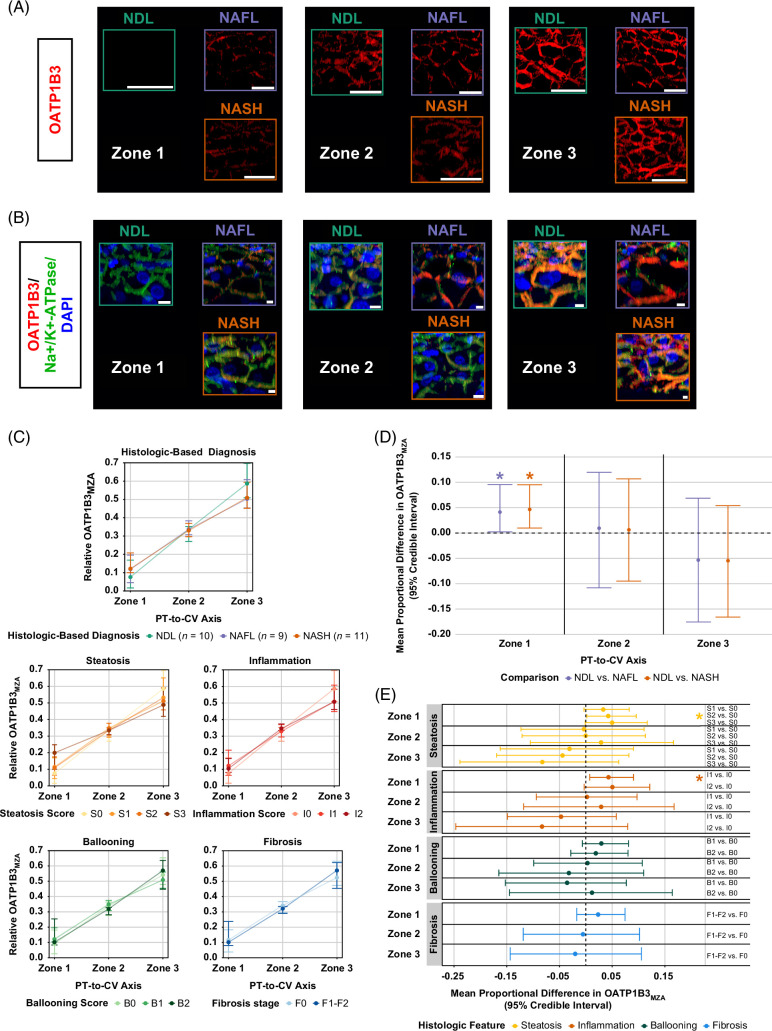
OATP1B3_MZA_ is altered in zone 1 in NAFL and NASH; increased zone 1 OATP1B3_MZA_ is associated with early-stage steatosis and lobular inflammation. (A, B) Representative three-dimensional images of OATP1B3 fluorescence surface masks used to assess (A) zonal abundance (scale bars, 50 μm) and (B) membrane localization. Yellow/orange coloration in masked fluorescence images indicates colocalization. Imaris software was used to quantify the volumetric overlap of surfaces. Scale bars, 20 μm. (C) Median (quartile 1; quartile 3) relative OATP1B3_MZA_ along the PT-to-CV axis grouped by NAFLD diagnosis or histologic score. Plotted are triplicate measures (where available) taken from different areas of each liver biopsy. Sample sizes for each histologic score are the same as detailed in the legend of Figure 2. (D, E) Mean differences in zonal OATP1B3_MZA_ for (D) NAFL and NASH compared to NDL biopsies and (E) various histologic scored biopsies compared to a score of 0 for the corresponding histologic category. Median and 95% credible intervals of each marginal distribution are shown. *95% credible interval does not cross 0. Abbreviations: CV, central vein; MZA, membrane-localized zonal abundance; NDL, non-diseased liver; OATP, organic anion transporting polypeptide; PT, portal tract.

Relative zonal OATP1B3 abundance (Figure 5A; Supplemental Figure S4C, http://links.lww.com/HC9/A781) and basolateral membrane localization (Figure 5B; Supplemental Figure S4D, http://links.lww.com/HC9/A781) measures were obtained across hepatic zones 1–3 for all available liver biopsies. These data (Figure 5C) were used for Dirichlet B-GLMMs. Periportal increases in OATP1B3_MZA_ were observed in NAFL and NASH biopsies; the mean differences compared to NDL biopsies were 0.041 and 0.047, respectively (Figure 5D). NAFLD diagnosis had a negligible impact on zone 2 and 3 OATP1B3_MZA_.

Periportal OATP1B3_MZA_ was increased in S2 and I1 biopsies, with a modest trend toward increasing periportal OATP1B3_MZA_ with steatosis and lobular inflammation severity (Figure 5E). The estimated mean differences of S2 and I1 for zone 1 OATP1B3_MZA_ when compared to S0 and I0 biopsies were 0.043 and 0.043, respectively. A negligible impact on zone 2 and zone 3 OATP1B3_MZA_ was observed for other histologic categories.

Minor changes were observed in periportal NTCP_MZA_ and OATP1B1_MZA_ in NAFLD (Supplemental Figure S5A, B, http://links.lww.com/HC9/A781). In NASH and I2 biopsies, there was a decrease in periportal NTCP_MZA_ compared to NDL and I0 biopsies, with estimated mean differences of −0.037 and −0.067, respectively (Supplemental Figure S5A, C, http://links.lww.com/HC9/A781). NAFL biopsies showed an increase in periportal OATP1B1_MZA_ with an estimated mean difference of 0.046 compared to NDL (Supplemental Figure S5B, http://links.lww.com/HC9/A781). However, B1 and B2 biopsies exhibited a decrease in periportal OATP1B1_MZA_ compared to B0, with respective estimated mean differences of −0.043 and −0.055 (Supplemental Figure S5D, http://links.lww.com/HC9/A781). Negligible differences were observed for zone 2 NTCP_MZA_ and OATP1B1_MZA_ across NAFLD diagnosis and histologic scoring groups.

## DISCUSSION

This study applied a novel machine learning image classification approach to obtain the first quantitative zonal abundance, plasma membrane localization, and MZA estimates for key hepatocyte BA transporters in NAFLD. By evaluating each outcome separately (i.e., zonal abundance and membrane localization), we provided new insight into the alterations of hepatocyte transporters during NAFLD progression. Furthermore, our transporter MZA data provide an additional explanation for previously reported clinical changes in NAFLD, such as altered BA homeostasis.

BSEP, NTCP, and OATP1B1, OATP1B3 zonal abundance patterns in NDL biopsies were consistent with previous studies indicating high pericentral OATP1B3 abundance, less preferential pericentral OATP1B1 abundance, and nonpreferential zonal distribution of BSEP and also NTCP (Figure 1).^22,31,32^ In our NASH cohort, periportal increases and pericentral decreases in BSEP abundance were observed. Although the observed periportal increases were slightly greater in magnitude than opposing pericentral decreases (0.058 vs. −0.042), this would theoretically result in a largely negligible net change in overall BSEP liver abundance, consistent with a previous report of no change in overall BSEP abundance in NAFLD measured by quantitative targeted absolute proteomics.^18^ These results support the validity of our approach in obtaining zonal data and demonstrate the value of zonal assessment to better understand NAFLD pathophysiology. Our finding of decreased pericentral BSEP abundance was corroborated by nonquantitative immunohistochemistry data that showed reduced BSEP abundance in regions with pronounced steatosis.^17^ It is reasonable to assume that these unspecified areas are located in the pericentral region because adult NAFLD is typically initiated through pericentral steatosis and inflammation. Isolated periportal disease is rare in adult patients.^35^ OATP1B1, OATP1B3 and NTCP overall abundance were decreased in 1 NASH cohort.^18^ Considering these data, increased relative periportal OATP1B3 in NAFLD and I1 biopsies indicates less zone 2 and 3 protein, suggesting a weaker pericentral gradient for OATP1B3 in NAFLD compared to NDL. No changes in OATP1B1 and NTCP zonal abundance in the present study suggest these proteins are equally downregulated across the acinus in NASH.

Negligible NAFLD-mediated changes in relative overall transporter plasma membrane localization were observed (Supplemental Figure S3, http://links.lww.com/HC9/A781). Interestingly, we observed decreased pericentral BSEP membrane localization and increased pericentral OATP1B3 membrane localization in NASH biopsies (Figure 3B). Furthermore, periportal and periportal/pericentral NTCP localization were decreased in B2 and F1-F2 biopsies, respectively (Figure 3C), suggesting a potential influence of advanced hepatocyte injury on NTCP membrane localization. Using qualitative imaging, Hardwick et al^29^ demonstrated cellular mislocalization of multidrug resistance–associated protein 2 (MRP2) in NASH, possibly due to reduced N-glycosylation, a critical mechanism for proper membrane trafficking. BSEP, NTCP, OATP1B1, and OATP1B3 are all glycosylated proteins with increased levels of their respective unglycosylated forms in NASH.^44^ However, the membrane localization of these proteins was differentially impacted across zones by NAFLD in our study suggesting that additional mechanisms are involved. Hepatocyte transporter membrane trafficking, insertion, and retrieval is a dynamic process involving multiple mechanisms^27^ that are altered in NASH.^45^ Another proposed hypothesis for NASH-mediated MRP2 internalization is oxidative stress.^45^ Oxidative stress is predominantly experienced by pericentral hepatocytes in NASH.^34^ It is possible that observed impairments in pericentral BSEP and NTCP localization are influenced by oxidative stress. The Rab11 pathway, which is involved in BSEP membrane retrieval, is also upregulated in NASH.^45^ Studies designed to investigate the precise mechanism(s) impacting transporter trafficking and localization in NAFLD across zonal regions are needed.

Our study revealed, for the first time, the important changes in BSEP_MZA_ associated with NAFLD. Specifically, increased periportal and decreased pericentral BSEP_MZA_ were observed in NASH and biopsies with higher histologic scores (Figure 4). Considering increases in systemic^10,11,13^, hepatic^12^, and intestinal^9,14^ BAs in patients with NASH, periportal hepatocytes are likely to encounter higher BA concentrations. Zone 1 is the predominant site of hepatocyte BA uptake in rats.^46^ Therefore, increased periportal BSEP_MZA_ may be a compensatory response to facilitate the excretion of excess BAs to avoid BA-induced hepatocyte injury in this region. Furthermore, periportal NTCP_MZA_ was decreased in NASH and I2 biopsies (Supplemental Figure S5, http://links.lww.com/HC9/A781), suggesting an additional adaptation to moderate hepatocyte BA exposure. Decreased pericentral BSEP_MZA_ in NASH biopsies and those of lower histologic scores (i.e., S1, I1, B1) suggest less BSEP functional capacity in zone 3 hepatocytes during the early stages of NASH. This finding aligns with reports of decreased pericentral bile canaliculi network connectivity, microcholestasis, and increased pericentral biliary pressure in early NASH.^6^ Reduced pericentral BSEP_MZA_ could also contribute to higher serum and liver BAs^6,9–14^ and pericentral injury^35^ in adults with NASH. FXR is preferentially active in the pericentral region^35^ and regulates BSEP expression.^27^ Therefore, the downregulation of FXR in NASH^47^ may contribute to lower pericentral BSEP_MZA_. Clinical improvements including lower gamma-glutamyl transferase levels with FXR agonist therapy in NASH fibrosis^48^ could involve FXR-mediated upregulation of pericentral BSEP to alleviate microcholestasis. However, the exact causative or compensatory nature of the observed NAFLD-associated changes in BSEP zonation and their role in clinical alterations of BA homeostasis require further investigation. Alterations in BSEP_MZA_ observed during liver biopsy assessment could serve as diagnostic and/or predictive biomarkers for NASH and/or NASH-related pharmacotherapies in the future.

Reduced BSEP function due to drug-mediated inhibition is one mechanism of DILI.^25^ Previous reports have suggested an increased DILI risk in the obese and NAFLD populations.^7^ The BSEP_MZA_ data obtained from our study offer insight into zonal regions potentially more susceptible to DILI in NAFLD. For example, decreased pericentral BSEP_MZA_ could predispose individuals to BA-mediated DILI in zone 3 as acute BA-mediated hepatocellular and cholestatic DILI always appear in this region.^49^ The BSEP_MZA_ data could be incorporated into quantitative systems toxicology models to optimize BSEP-mediated DILI predictions in the metabolic syndrome population.^25^

We also observed increased periportal OATP1B3_MZA_ in NAFLD (Figure 5). While the mechanism(s) behind changes in OATP1B3_MZA_ and increased periportal OATP1B1_MZA_ in NAFL (Supplemental Figure S5B, http://links.lww.com/HC9/A781) are unknown, the liver can undergo remodeling resulting in zonal changes in protein expression during NAFLD progression.^36^ Computational modeling incorporating the data generated could explain clinical pharmacokinetic observations from previous studies. As a proof of concept, OATP1B1 zonal abundance data derived from our study were incorporated into a physiologically based multicompartment liver model to successfully recapture concentration-time profiles of a complex transporter-mediated drug-drug interaction scenario involving repaglinide and rifampin in a NDL population.^50^ Our current dataset would allow for further simulation of drug response and toxicity in a physiologically based pharmacokinetic/pharmacodynamic or quantitative systems toxicology framework for both NDL and NAFLD patient populations.

Considerable variability was observed within this clinical cohort, particularly in histologic scoring groups with smaller sample sizes. However, our primary conclusions focus on results with 95% Bayesian credible intervals that do not cross zero, ensuring at least a 95% probability of true differences. Some results with notable variability where the 95% credible interval marginally crossed zero were interpreted as having no significant difference. In these cases, it is theoretically plausible, albeit with <95% certainty, that a true difference exists. While this approach limited our ability to make definitive conclusions for some findings, it ensured statistical robustness, consistency, and higher confidence in our main conclusions. A limitation of this study was that the functional significance of the findings in this clinical cohort was not directly assessable. Specifically, we were unable to evaluate the presence of microcholestasis or elevated serum BAs due to incomplete data on relevant biomarkers, such as gamma-glutamyl transferase and alkaline phosphatase, and serum samples were not available to quantify BA concentrations. Consequently, we relied on prior observations from similar cohorts with NAFLD to contextualize our results. Furthermore, the number of available liver biopsy samples per patient was limited, and we were able to assess only one transporter and membrane marker per sample. Ideally, a comprehensive examination of hepatocyte transporters involved in hepatic BA disposition would have included basolateral efflux transporters (eg, MRP3, MRP4, and organic solute transporterα/β) that are upregulated in patients with NAFLD^23^ and may also contribute to previously observed increases in serum BAs. Future studies to quantify changes in the plasma membrane localization and zonal abundance of these transporters in NAFLD are warranted.

Collectively, these data shed light on NAFLD pathophysiology and offer new insight into the potential for alterations in drug disposition and BA homeostasis during NAFLD progression. Our findings also improve our understanding of the impact of NAFLD on zonal abundance and cellular localization of hepatocyte BA and drug transporters. This study emphasizes the need for further investigation to elucidate the role of BSEP in NAFLD progression and pharmacologic outcomes.

## Supplementary Material
