# Supplementary material for: Alterations in zonal distribution and plasma membrane localization of hepatocyte bile acid transporters in patients with NAFLD
Source: Hepatol Commun. 2024 Feb 14;8(3):e0377. doi: 10.1097/HC9.0000000000000377 (PMC10871794; doi:10.1097/HC9.0000000000000377)
Supplement: Supplementary file 1 [file hc9-8-e0377-s001.docx]

**Alterations in Zonal Distribution and Plasma Membrane Localization of Hepatocyte Bile Acid Transporters in NAFLD Patients**

William A. Murphy, PharmD^1^, Anna Mae Diehl, MD^2^, Matthew S. Loop, PhD^3^, Dong Fu, PhD^1^, Cynthia D. Guy, MD^4^, Manal F. Abdelmalek, MD, MPH^5^, Georgia Sofia Karachaliou, MD, MSc^2^, Noora Sjöstedt, PhD^6^, Sibylle Neuhoff, PhD^7^, Paavo Honkakoski, PhD^1,8^, Kim L.R. Brouwer, PharmD, PhD^*1^

^1^Division of Pharmacotherapy and Experimental Therapeutics, UNC Eshelman School of Pharmacy, University of North Carolina at Chapel Hill, Chapel Hill, North Carolina, USA

^2^Division of Gastroenterology and Hepatology, Duke University Medical Center, Durham, North Carolina, USA

^3^Health Outcomes Research and Policy, Harrison College of Pharmacy, Auburn University, Auburn, Alabama, USA

^4^Department of Pathology, Duke University, Durham, North Carolina, USA

^5^Division of Gastroenterology and Hepatology, Mayo Clinic, Rochester, Minnesota, USA

^6^Division of Pharmaceutical Biosciences, Faculty of Pharmacy, University of Helsinki, Helsinki, Finland

^7^Certara UK Ltd. (Simcyp division), Sheffield, United Kingdom

^8^School of Pharmacy, University of Eastern Finland, Kuopio, Finland

**Address for Correspondence:**

Kim L.R. Brouwer

Division of Pharmacotherapy and Experimental Therapeutics

UNC Eshelman School of Pharmacy

100 L Beard Hall, CB# 7569

Chapel Hill, NC 27599‑7569, USA

Email: [kbrouwer@unc.edu](mailto:kbrouwer@unc.edu)

^*^The current affiliation for Dong Fu is: Tessera Therapeutics, 101 South Street, Somerville, MA 02143.

**Supplemental Methods**

**Reagents**

Citrate Buffer (pH 6.0) 10x Antigen Retriever and bovine serum albumin (BSA) were purchased from Millipore Sigma (Burlington, MA). Goat serum and Fluoromount-G Mounting Medium were purchased from Southern Biotech (Birmingham, AL), and 4′,6-diamidino-2-phenylindole (DAPI) solution (1 mg/mL) was purchased from Fisher Scientific (Waltham, MA).

**Liver Biopsy Preparation**

Percutaneous liver needle biopsies obtained from patients who underwent a standard of care liver biopsy for evaluation of presumed NAFLD at Duke University were immediately fixed in formalin, embedded in paraffin, and stored in the Duke NAFLD Clinical Database & Biorepository. Formalin-fixed paraffin-embedded (FFPE) biopsy samples were cut into 10-μm sections and placed on electrostatically charged glass slides.

**Fluorescence Immunohistochemistry**

In preparation for fluorescence immunohistochemical staining, FFPE slides were deparaffinized with xylenes, rehydrated using a gradually decreasing ethanol gradient of 100% to 50%, and then permeabilized in Tris Buffered Saline with Tween-20 (TBS-T). For heat-induced antigen retrieval, samples underwent two separate heating cycles in a domestic microwave oven (5 min at 100% power [1650-watts] followed by 20 min at 50% power [825-watts]) while completely submerged in 1x Antigen Retriever citrate buffer (pH 6.0). After cooling, slides were briefly re-permeabilized in TBS-T and then blocked using 1% w/v BSA and 10% v/v goat serum in TBS-T for 1 hr at room temperature. After blocking, samples were incubated overnight at 4°C in a 1% BSA solution containing two separate diluted primary antibodies; one targeting a single transport protein of interest (mouse anti-BSEP; rabbit anti-NTCP; mouse anti-OATP1B1; rabbit anti-OATP1B3) and one targeting an appropriate membrane marker (mouse anti-Na^+^/K^+^-ATPase or rabbit anti-Na^+^/K^+^-ATPase for basolateral transporters[^1^](https://sciwheel.com/work/citation?ids=15461983&pre=&suf=&sa=0&dbf=0) (*i.e.*, OATP1B1, OATP1B3, NTCP); rabbit anti-aminopeptidase N (CD13) for BSEP [canalicular transporter[^1^](https://sciwheel.com/work/citation?ids=15461983&pre=&suf=&sa=0&dbf=0)]) (**Table 2**). The following day, samples were rinsed with 10x TBS solution and incubated for 1 hr at room temperature in a 1% BSA solution containing fluorescence-dye conjugated anti-mouse (Alexa Fluor 647) and anti-rabbit (Alexa Fluor 568) secondary antibodies. Fluorescence-dye conjugated secondary antibodies with peak emission within the 400-550 nm range (e.g., Alexa Fluor 488) were avoided to minimize undesirable capture of hepatic auto-fluorescence during imaging[^2,3^](https://sciwheel.com/work/citation?ids=13390789,13391590&pre=&pre=&suf=&suf=&sa=0,0&dbf=0&dbf=0). After washing with TBS, samples were stained with DAPI (1 μg/mL) for 5 min at room temperature to visualize hepatocyte nuclei, again washed with TBS, and then mounted with a glass cover slip using Fluoromount-G Mounting Medium. Samples were stored in a dry, dark location and imaged between 2 days and 1.5 months after mounting. In-house data suggest fluorescence stability of similar samples (FFPE human liver) for at least 1 year when stored under dark, dry conditions.

*Isotype Control Antibody Testing*

All isotype control antibody tests (**Table S1**), when using the same secondary antibody and imaging at the same laser power/gain range corresponding to the respective image acquisition of each primary antibody, showed negligible background signal/autofluorescence apart from that relating to the OATP1B1 primary antibody. Correction for observed background signal/autofluorescence was performed by increasing the minimum voxel size to 500 and lowering the background subtraction maximum diameter to 0.75 μm during volumetric surface rendering of OATP1B1 in Imaris Image Analysis software (**Table S2**).

**Table S1.** Description of isotype antibodies used for negative control testing in this study. Tested dilutions were determined to match molar concentration of corresponding primary antibody.

| **Control Antibody**  **(Host species-target)** | **Catalog number** | **Supplier** | **Tested Dilution(s)** | **Corresponding Primary Antibody(ies)**  **(Catalog #)** | **Secondary Antibody** |
| --- | --- | --- | --- | --- | --- |
| Mouse anti-IgG_2a_ kappa Isotype Control | 14-4724-81 | Invitrogen (Waltham, MA) | 1:100 | BSEP (sc-74500) | Goat-anti-mouse IgG AlexaFluor 647 |
| Rabbit-anti-IgG Isotype Control | MA5-16384 | Invitrogen | 1:30/1:1,500 | NTCP (GTX17693); OATP1B3 (custom-made); CD13 (ab108382) | Goat-anti-rabbit IgG AlexaFluor 568 |
| Mouse-anti-IgM Isotype Control | 14-4752-81 | Invitrogen | 1:100 | OATP1B1 (NB10074481) | Goat-anti-mouse IgG AlexaFluor 647 |
| HRP Conjugated Rabbit-anti-IgG Isotype Control | bs-0295P-HRP | Bioss Antibodies (Woburn, MA) | 1:1,000 | Na^+^/K^+^-ATPase (ab185065) | Goat-anti-rabbit IgG AlexaFluor 568 |
| Mouse anti-IgG_2b_ kappa Isotype Control | 14-4732-81 | Invitrogen | 1:1,200 | Na^+^/K^+^-ATPase  (sc-48345) | Goat-anti-mouse IgG AlexaFluor 647 |

**Table S2.** Imaris Image Analysis software parameters used for surface rendering.

| **Protein Marker** | **Smoothing Diameter (μm)** | **Background Subtraction Diameter (μm)** | **Minimum Voxel Size** |
| --- | --- | --- | --- |
| BSEP | 0.208 | 1.2 | 10 |
| CD13 | 0.400 | 1.2 | 10 |
| Na^+^/K^+^-ATPase | 0.208 | N/A | 100 |
| NTCP | 0.208 | 1.0 | 10 |
| OATP1B1 | 0.208 | 0.75 | 500 |
| OATP1B3 | 0.208 | 1.2 | 10 |

*Primary Antibody Validation*

Dilution factors for each primary antibody were initially informed by manufacturer recommendations and then optimized using HuH-7 cells[^4^](https://sciwheel.com/work/citation?ids=13534038&pre=&suf=&sa=0&dbf=0) and FFPE human liver biopsy samples obtained from the NIH Liver Tissue Distribution System (University of Minnesota collection center). The OATP1B3 primary antibody was custom-made, with incubation concentrations informed by previous studies[^5,6^](https://sciwheel.com/work/citation?ids=4907,11156609&pre=&pre=&suf=&suf=&sa=0,0&dbf=0&dbf=0) and optimized using the same samples mentioned above.

**Confocal Microscopy Image Acquisition**

Image acquisition was performed with Zeiss Zen software (version 2.3 SP1) via a Zeiss LSM 880 confocal laser scanning microscope (Carl Zeiss AG, Oberkochen, Germany) using an EC Plan-Neofluar 40x/1.30 Oil DIC M27 objective. Images were acquired using 405 nm (DAPI), 561 nm (Alexa Fluor 568), and 633 nm (Alexa Fluor 647) laser lines in a 1x1 binning mode with Airyscan off. To avoid any potential overlap across fluorescence channels during acquisition, detection wavelengths for each fluorophore were as follows: DAPI, 444-478 nm; Alexa Fluor 568, 570-633 nm; Alexa Fluor 647, 638-703 nm. Different laser powers and gain were used for each sample to optimize image clarity. These differences did not impact volumetric surface rendering in Imaris, nor the quantitative assessment of plasma membrane localization or zonal abundance as these parameters were assessed in a relative, not absolute, manner. Scaling per pixel (x-y-z resolution) was as follows 0.208 μm-0.208 μm-0.55 μm. A pinhole size of 43.7 μm was used for all channels to ensure the same Z axis quality was acquired for each channel.

**Image Analysis – Plasma Membrane Localization Quantification**

For each transporter of interest, 3D-surface rendering was performed using a smoothing parameter, background subtraction with automatic thresholding, and voxel size limits (see **Table S2** for specific parameters) to selectively capture fluorescence signals of interest. The Imaris Machine Learning Classifier was used to segment out non-hepatocyte (e.g., sinusoidal cavity) surfaces. Manual identification of non-parenchymal vs. parenchymal surfaces was performed at random areas within the region of interest (ROI), and then used as training data for the machine learning algorithm. Manual selection was used solely for samples in instances where the machine learning classifier did not adequately segment out non-hepatocyte regions.

Surface rending for the basolateral membrane marker (Na^+^/K^+^-ATPase) was initiated using a smoothing parameter of 0.208 μm. The smoothed fluorescence source channel corresponding to the marker was then exported to Fiji Labkit (ImageJ version 1.53t)[^7^](https://sciwheel.com/work/citation?ids=12654452&pre=&suf=&sa=0&dbf=0) for segmentation using a random forest-based pixel classification algorithm. Intracellular and other non-membranous pixels were selected manually as “background”, while plasma membrane pixels were selected as “foreground” at random slices and areas within the ROI. The Labkit Pixel Classifier was then trained using 3D dimensions with default sigma values (1.0; 2.0; 4.0; 8.0) and structure tensor eigenvalues for each sigma. The segmentation prediction was then computed and sent back to the Imaris Image Analysis platform, with a final machine learning classification step to remove any non-membranous surfaces.

Surface rendering for the canalicular marker (CD13) was performed in Imaris as described above for the transporters using a smoothing parameter of 0.400 μm (see **Table S2** for additional parameters). A higher smoothing parameter was used for the CD13 surface compared to the Na^+^/K^+^-ATPase surface. This adjustment was performed to more effectively integrate regions of dysconnectivity observed with the canalicular staining pattern of the CD13 marker. Surface classification for CD13 involved selection of membrane vs. non-membrane surfaces, rather than non-hepatocyte vs. hepatocyte affiliated surfaces.

**Bayesian Regression Modeling**

Bayesian regression models were developed to assess each transporter of interest in relation to NAFLD diagnosis (**Figures 1C, 3B**) and histologic score (**Figures 2, 3C**). All Bayesian models were compiled and run using the *brms* package (v.2.19.0) in RStudio[^8^](https://sciwheel.com/work/citation?ids=4604908&pre=&suf=&sa=0&dbf=0). All continuous fixed effect variables (i.e., age, BMI) were median centered prior to modeling. Model outputs and diagnostic measures (e.g., posterior predictive checks) for all skew-normal and Beta family models were assessed using the *bayesplot* package (v.0.13.1) in RStudio. Dirichlet family model output and posterior predictive checks were evaluated using the *tidybayes* package (v.3.0.4) in tandem with user-generated R code. All user-defined prior distributions are listed in **Table S3**; model parameters not listed in this Table used the default prior distributions in the *brms* package for the respective model family. Beta models for the zonal membrane localization outcome included a random intercept on sample ID and NAFLD diagnosis or histologic feature as a covariate on the phi (precision) parameter. Beta models assessing overall membrane localization as the outcome of interest did not specify a formula on the phi parameter due to issues with model convergence when including a formula for phi. Dirichlet models did specify a phi parameter due to complexity of the corresponding proportional outcome of interest compounded by the additional fixed effects already included in the model. The skew-normal model family was only used when modeling the hepatic lobule radius outcome. A random seed of 1234 was used for all Monte Carlo simulations involving Bayesian model runs and marginal mean calculations; 4,000 Monte Carlo Markov Chain samples across 4 chains with 2,000 warmup samples were used for all models. All observed Rhat values were below 1.01 with effective sample sizes greater than 1,000. The *ggplot* package (v.3.4.2) was used to generate all posterior distribution and marginal mean effect size figures. Marginal mean estimates for skew-normal and Beta regression models were obtained using the *emmeans* package (v.1.8.6); marginal means for Dirichlet regression models were obtained using the *tidybayes* package (v.3.0.4) alongside user-generated R code. Estimated mean differences for histologic based diagnoses were calculated by comparing NAFL and NASH biopsies to NDL biopsies. Estimated mean differences for histologic scoring were calculated by comparing biopsies of various histologic scores to a score of 0 within the corresponding histologic category. All predicted marginal mean effect sizes are for a hypothetical subject of our median sample age (50 years) and body-mass index (BMI; 33.56 kg/m^2^) and averaged over all available levels of race and gender. All marginal mean effect sizes were reported using the median point estimate and 95% credible interval.

**Table S3.** Bayesian regression model general parameterizations and priors for each outcome assessed using the *brms* package (v. 2.18.0) in R (v. 4.3).

| **Outcome** | **Model Family** | **Predictor(s) of Interest** | **Fixed Effects** | **Random Effects** | **User-specified Priors** | **Phi Parameters** | **Interaction Term** |
| --- | --- | --- | --- | --- | --- | --- | --- |
| Relative Zonal Abundance | Dirichlet | NAFLD diagnosis or Histologic feature | ***NAFLD diagnosis or Histologic feature***; BMI; Age; Gender; Race | Sample ID | student_t(3,0,2.5) for intercept;  normal(0,1) for fixed effects^1^ | None | None |
| Overall Plasma Membrane Localization | Beta | NAFLD diagnosis or Histologic feature | ***NAFLD diagnosis or Histologic feature***; BMI; Age; Gender; Race | Sample ID | student_t(3,0,2.5) for intercept, random effects, and random effects intercept grouped by Sample ID;  normal(0,1) for fixed effects | None | None |
| Zonal Plasma Membrane Localization | Beta | NAFLD diagnosis or Histologic feature | ***NAFLD diagnosis or Histologic feature***; BMI; Age; Gender; Race | Sample ID | student_t(3,0,2.5) for intercept and Phi intercept; normal(0,1) for fixed effects and Phi fixed effects | Sample ID (Random Effect); NAFLD diagnosis or Histologic feature (Fixed Effect) | NAFLD Diagnosis or Histologic feature * Zone |
| Membrane-Localized Zonal Abundance (MZA) | Dirichlet | NAFLD diagnosis or Histologic feature | ***NAFLD diagnosis or Histologic feature***; BMI; Age; Gender; Race | Sample ID | student_t(3,0,2.5) for intercept;  normal(0,1) for fixed effects^2^ | None | None |
| Hepatic Lobule Radius | Skew-normal | NAFLD diagnosis | ***NAFLD diagnosis***; BMI; Age; Gender; Race | Sample ID | Normal(600,100) for intercept;  Normal(0,3) for random effects | N/A | None |

BMI, body-mass index

^1^A student-t prior with a distribution of (3, 0.5, 2.5) was also included for steatosis and fibrosis models on random effect intercept grouped by sample ID for relative Zone 3 abundance

^2^A student-t prior with a distribution of (3, 0.5, 2.5) was also included for hepatocellular ballooning models on random effect intercept grouped by sample ID for Zones 2 and 3 MZA

**Supplemental Results**

***Hepatic Lobule Radius is Increased in NAFL and NASH***

The x-axis dimension (length) for each ROI spanned from the midpoint of the CV to the edge of the connective tissue surrounding the PT (beginning of portal parenchyma) and was used to estimate the hepatic lobule radius (**Figures S1A-B**). When adjusting for demographic variables and inter-individual variability across measurements, the B-GLMM estimated mean increases in lobule radius were 50.3 μm (4.3­—99.1 μm) [median point estimate (95% credible interval)], 65.0 μm (22.9—109.0 μm), and 14.6 μm (-25.2—54.3 μm) during progression from NDL to NAFL, NDL to NASH, and NAFL to NASH, respectively (**Figure S1C**). Hepatic lobule radii measurements and observed changes in NAFLD aligned with previous studies[^9,10^](https://sciwheel.com/work/citation?ids=8201513,5118494&pre=&pre=&suf=&suf=&sa=0,0&dbf=0&dbf=0).


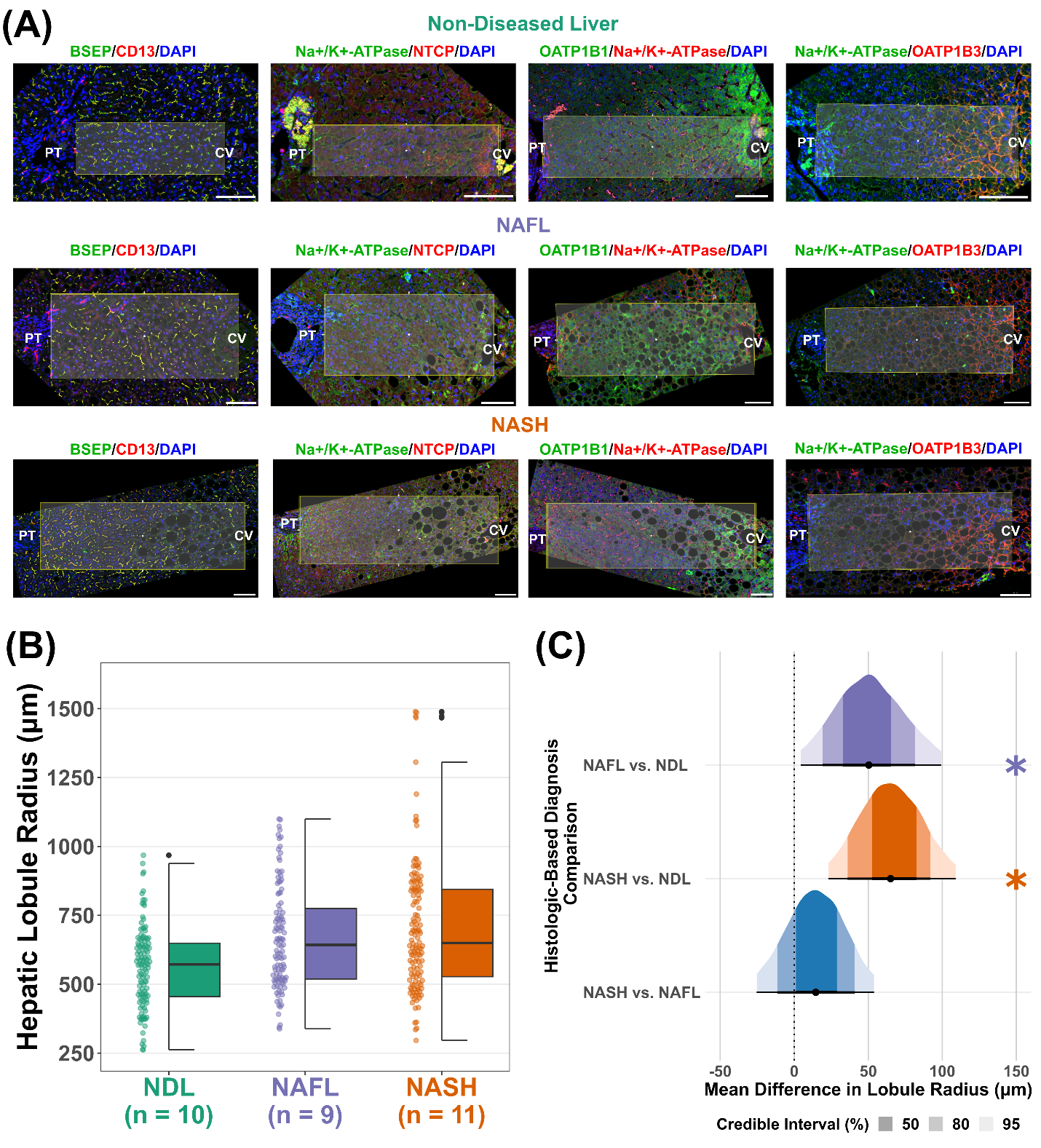


**Figure S1. Hepatic lobule radius is increased in NAFLD; no difference in hepatic lobule radius is observed between NAFL and NASH.** (A) Representative images of liver biopsy samples from subjects with Non-Diseased Liver (NDL), NAFL, and NASH are shown. Hepatic acinus size/lobule radius was measured from the edge of the portal triad (PT) to the midpoint of the central vein (CV). Measurement area was defined using a rectangular user-defined region of interest in Imaris Image Analysis software. *Scale bars, 100 μm*. Panel (B) depicts all hepatic acinus radius measurements across NDL, NAFL, and NASH biopsy samples. Triplicate measurements were taken within each sample (except for one NDL biopsy). Each subject had four samples evaluated (1 for each transporter of interest). Boxplots depict median and quartiles 1-3; upper and lower whiskers represent maximum and minimum values. Outliers that exceed 1.5 times the interquartile range are plotted as black points. Plotted are replicate measures taken from different areas within each liver biopsy. Four separate biopsies from each subject were evaluated (one per transport protein of interest). Statistical testing was not performed on the raw data. All available measures were used for Bayesian regression analysis. (C) Mean posterior predicted difference in hepatic lobule radius for NAFL and NASH biopsies compared to NDL; and NASH compared to NAFL biopsies. A Bayesian skew-normal family regression model was developed and included a random intercept on biopsy sample ID to account for interindividual variability. The plotted median point estimates and credible intervals are for a single typical hypothetical subject of median sample age (50 years) and body-mass index (BMI; 33.56 kg/m^2^) averaged over all levels of gender and race. **95% credible interval does not cross 0*.

**
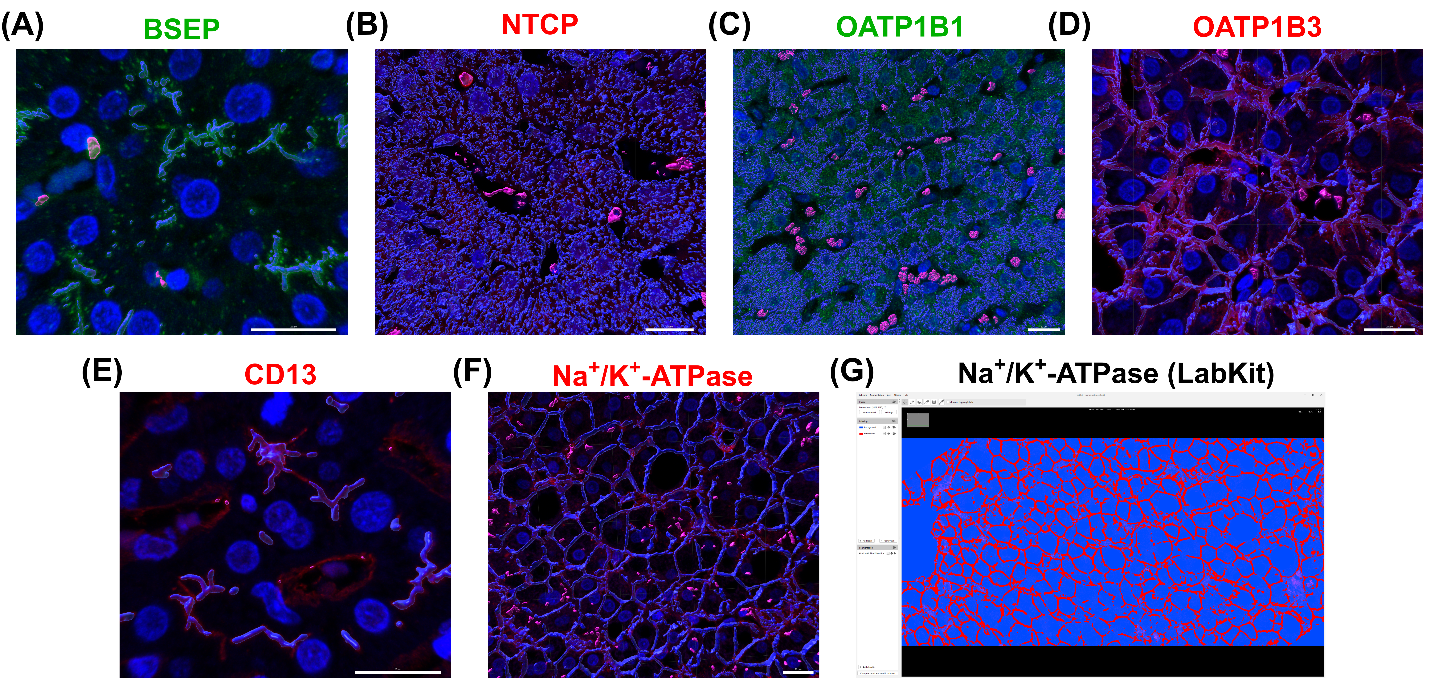
**

**Figure S2.** **Use of Imaris machine learning classification and Fiji Labkit to segment transporter and membrane marker volumetric surfaces derived from fluorescence data.** (A-F) Imaris machine learning image classification to segment fluorescence signals of interest for transporter and plasma membrane markers prior to membrane localization analysis. Nuclei demonstrate blue fluorescence signal. Transporters/membrane markers portray red or green fluorescence signal depending on the secondary antibody and corresponding image acquisition parameters. Blue volumetric surfaces were those identified as foreground (parenchymal/plasma membrane); pink volumetric surfaces are those identified as background (non-parenchymal/non-plasma membrane) by the Imaris machine learning classifier. Background surfaces were subsequently filtered out to allow for proper assessment of the foreground/surfaces of interest. *Scale bars, 20 μm*. (G) Fiji Labkit was used in conjunction with the Imaris platform to perform automated segmentation of fluorescence signal associated with Na^+^/K^+^ ATPase (red color indicates foreground, blue indicates background). Foreground/signal of interest was used to develop the final basolateral membrane marker surface in Imaris.

***OATP1B3 Overall Plasma Membrane Localization is Increased in Biopsies with a Steatosis Score of 2***

Beta B-GLMMs were applied to quantitatively estimate the specific impact of histologic-based NAFLD diagnosis and histologic features on overall hepatocyte transporter membrane localization while adjusting for inter-individual variability across measurements. While there were negligible impacts of NAFLD on overall membrane localization of BSEP, NTCP, and OATP1B1, a modest trend towards increased OATP1B3 membrane localization was observed with NAFLD progression (**Figure S3A**). Furthermore, S2 biopsies showed an increase in OATP1B3 membrane localization relative to S0 biopsies. The estimated mean effect sizes on OATP1B3 localization for NASH compared to NDL and S2 compared to S0 biopsies expressed as percentage points were respectively 5.9 (-0.6 – 10.6) [median (95% credible interval)] and 7.0 (0.5 – 14.9) (**Figure S3B**). OATP1B1 membrane localization trended higher with increasing steatosis and lobular inflammation severity; however, the 95% credible interval of these estimated mean differences crossed 0% (**Table S5**).


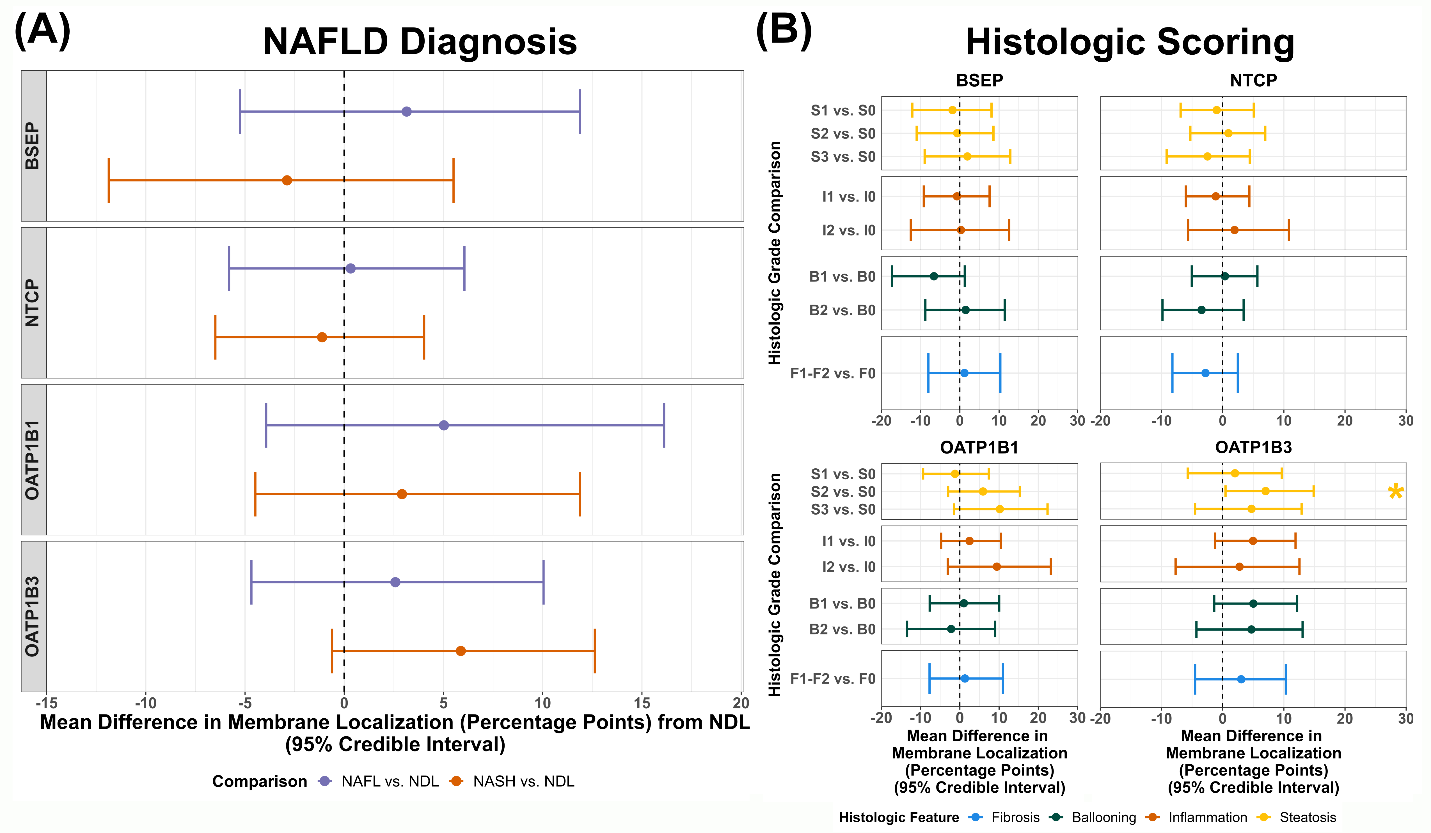


**Figure S3. Bayesian modeling of overall transporter membrane localization by NAFLD diagnosis and histologic features.** Mean posterior predicted difference of relative transporter membrane localization as a pooled measure across the hepatic acinus for NAFL and NASH biopsies compared to Non-Diseased Liver (NDL) biopsies and various histologic scores compared to a score of 0 for the corresponding histologic category. Bayesian Beta family regression models were developed for each transporter of interest; all models included a random intercept on biopsy sample ID to account for intra-/interindividual variability. The plotted median point estimates and 95% credible intervals are for a single typical hypothetical subject of median sample age (50 years) and body-mass index (BMI; 33.56 kg/m^2^) averaged over all levels of sex and race. The sample sizes for each NAFLD diagnosis group are as follows: NDL (*n*=10), NAFL (*n*=9), NASH (*n*=11). The sample sizes for each histologic score are as follows: S0 (*n*=10), S1 (*n*=7), S2 (*n*=8), S3 (*n*=5); I0 (*n*=10), I1 (*n*=17), I2 (*n*=3); B0 (*n*=20), B1 (*n*=7), B2 (*n*=3); F0 (*n*=25), F1-F2 (*n*=5). **95% credible interval does not cross 0*.


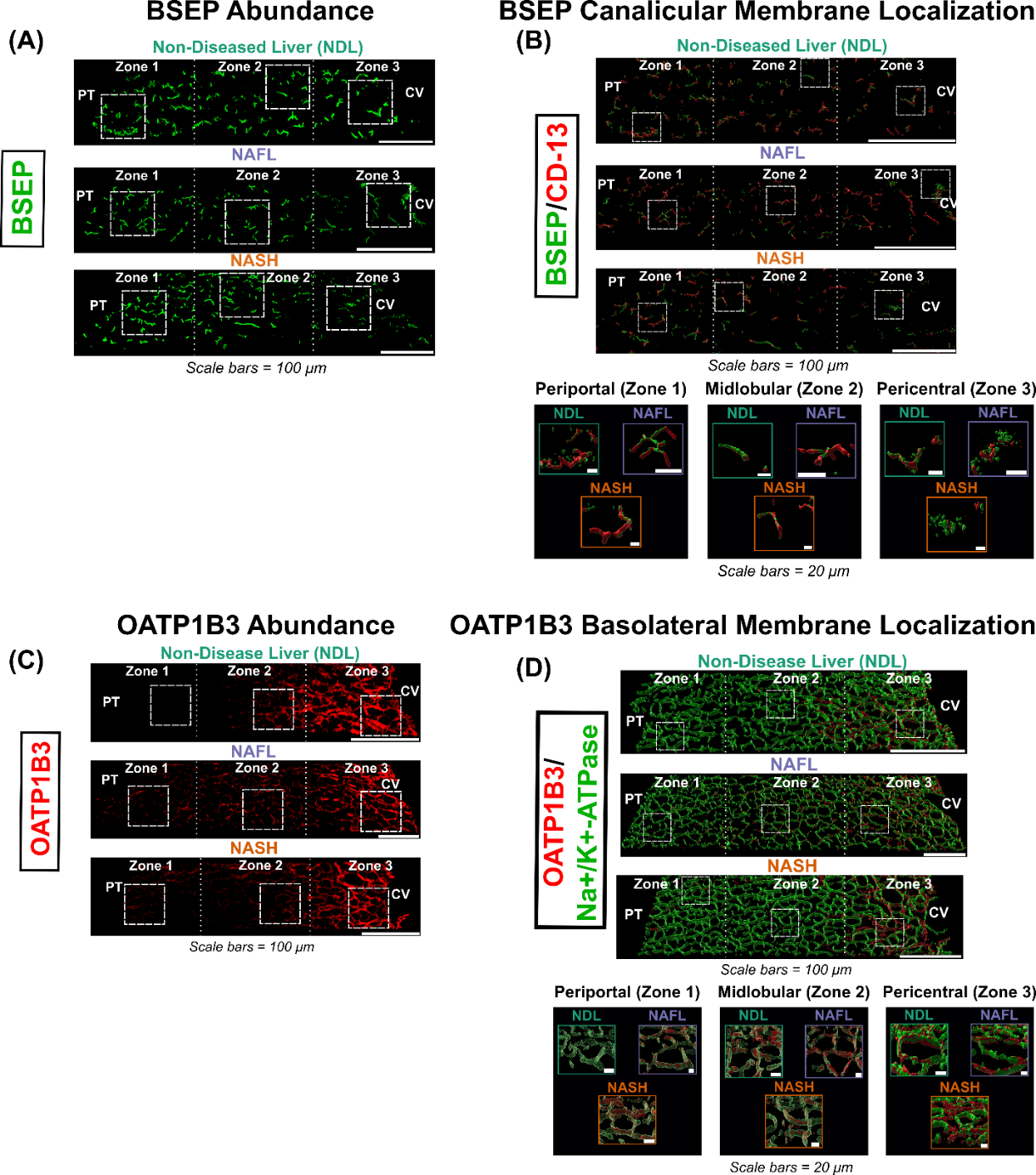


**Figure S4. (A-B) Original representative images used for assessment of BSEP_MZA_ (framed portions were enlarged and depicted in Figure 4A-B) and (C-D) OATP1B3_MZA_ (****framed portions were enlarged and depicted in Figure 5A-B**)**.** **Panels B and D were depicted as masked fluorescence images in Figures 4B and 5B, respectively, and are shown here as 3D volumetric surfaces rendered by Imaris Image Analysis software to properly assess transporter plasma membrane localization.**


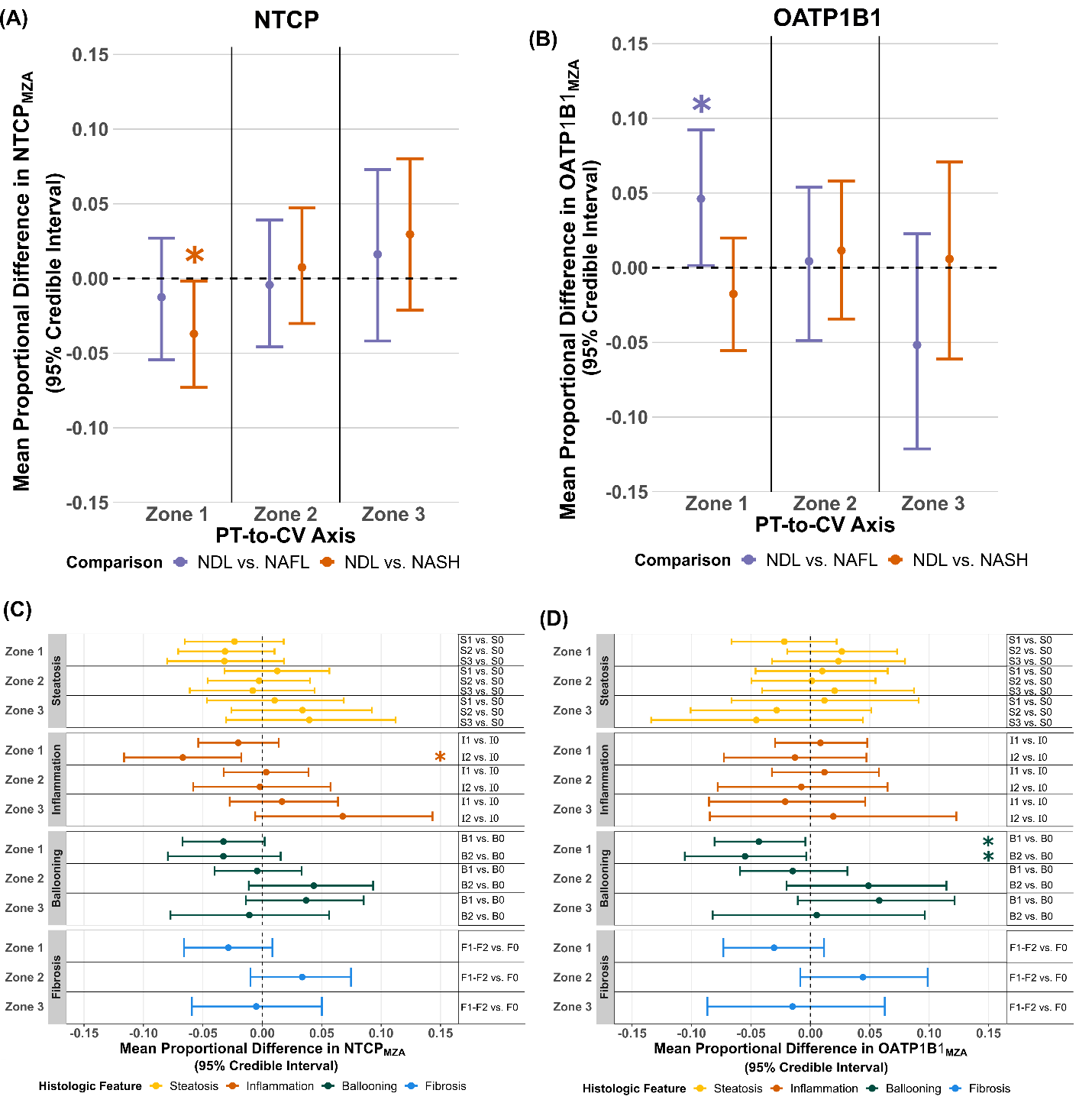


**Figure S5. Bayesian regression output of NTCP_MZA_ and OATP1B1_MZA_ by NAFLD diagnosis and histologic features.** (A,C) Mean differences in zonal NTCP_MZA_ and OATP1B1_MZA_ (B,D) for (A,B) NAFL and NASH compared to Non-Diseased Liver (NDL) biopsies and (C,D) various histologic scored biopsies compared to a score of 0 for the corresponding histologic category. Bayesian regression models were developed to assess the impact of histologic-based NAFLD diagnosis and histologic features on relative NTCP_MZA_ and OATP1B1_MZA_. Median point estimates and 95% credible intervals of each marginal distribution are shown. The sample sizes for each NAFLD diagnosis group are as follows: NDL (*n*=10), NAFL (*n*=9), NASH (*n*=11). The sample sizes for each histologic score are as follows: S0 (*n*=10), S1 (*n*=7), S2 (*n*=8), S3 (*n*=5); I0 (*n*=10), I1 (*n*=17), I2 (*n*=3); B0 (*n*=20), B1 (*n*=7), B2 (*n*=3); F0 (*n*=25), F1-F2 (*n*=5). **95% credible interval does not cross 0*.

**Table S4.** Estimated mean differences (median point estimates and 95% credible intervals) for relative zonal abundance Dirichlet family Bayesian models.

|  | | *NAFLD Diagnosis* | | *Steatosis* | | *Lobular Inflammation* | | *Hepatocellular Ballooning* | | *Fibrosis* | |
| --- | --- | --- | --- | --- | --- | --- | --- | --- | --- | --- | --- |
| **Transporter** | **Zone** | **Comp** | **Median (95% CI)** | **Comp** | **Median (95% CI)** | **Comp** | **Median (95% CI)** | **Comp** | **Median (95% CI)** | **Comp** | **Median (95% CI)** |
| BSEP | 1 | NAFL vs. NDL | 0.020  (-0.0101 – 0.049) | S1 vs. S0 | 0.066*  (0.038 – 0.096) | I1 vs. I0 | 0.045*  (0.019 – 0.072) | B1 vs. B0 | 0.050*  (0.024 – 0.077) | F1-F2 vs. F0 | 0.038*  (0.0075 – 0.069) |
| BSEP | 1 | NASH vs. NDL | 0.058*  (0.032 – 0.085) | S2 vs. S0 | 0.014  (-0.014 – 0.043) | I2 vs. I0 | 0.039  (-0.0013 – 0.080) | B2 vs. B0 | 0.054*  (0.018 – 0.091) |  | |
| BSEP | 1 |  | | S3 vs. S0 | 0.054*  (0.018 – 0.090) |  | |  | |  |  |
| BSEP | 2 | NAFL vs. NDL | -0.0024  (-0.034 – 0.027) | S1 vs. S0 | -0.016  (-0.045 – 0.014) | I1 vs. I0 | -0.010  (-0.037 – 0.017) | B1 vs. B0 | -0.012  (-0.039 – 0.015) | F1-F2 vs. F0 | -0.013  (-0.043 – 0.018) |
| BSEP | 2 | NASH vs. NDL | -0.016  (-0.043 – 0.012) | S2 vs. S0 | -0.0019  (-0.031 – 0.027) | I2 vs. I0 | -0.015  (-0.058 – 0.025) | B2 vs. B0 | -0.022  (-0.059 – 0.014) |  | |
| BSEP | 2 |  | | S3 vs. S0 | -0.021  (-0.056 – 0.015) |  | |  | |  |  |
| BSEP | 3 | NAFL vs. NDL | -0.017  (-0.054 – 0.022) | S1 vs. S0 | -0.050*  (-0.088 to 0.016) | I1 vs. I0 | -0.035*  (-0.069 to -0.0017) | B1 vs. B0 | -0.038*  (-0.072 to -0.0051) | F1-F2 vs. F0 | -0.025  (-0.063 – 0.014) |
| BSEP | 3 | NASH vs. NDL | -0.042*  (-0.076 to -0.0098) | S2 vs. S0 | -0.0121  (-0.047 – 0.023) | I2 vs. I0 | -0.024  (-0.073 – 0.031) | B2 vs. B0 | -0.032  (-0.076 – 0.014) |  | |
| BSEP | 3 |  | | S3 vs. S0 | -0.032  (-0.076 – 0.0090) |  | |  | |  |  |
| NTCP | 1 | NAFL vs. NDL | -0.00954  (-0.035 – 0.015) | S1 vs. S0 | -0.00015  (-0.024 – 0.025) | I1 vs. I0 | -0.0043  (-0.025 – 0.016) | B1 vs. B0 | 0.0036  (-0.019 – 0.026) | F1-F2 vs. F0 | -0.013  (-0.037 – 0.011) |
| NTCP | 1 | NASH vs. NDL | -0.0086  (-0.030 – 0.013) | S2 vs. S0 | -0.015  (-0.040 – 0.0084) | I2 vs. I0 | -0.034*  (-0.066 to -0.0029) | B2 vs. B0 | -0.0072  (-0.037 – 0.023) |  | |
| NTCP | 1 |  | | S3 vs. S0 | -0.017  (-0.048 – 0.013) |  | |  | |  |  |
| NTCP | 2 | NAFL vs. NDL | -0.0059  (-0.031 – 0.019) | S1 vs. S0 | -0.0071  (-0.031 – 0.019) | I1 vs. I0 | -0.0054  (-0.025 – 0.016) | B1 vs. B0 | -0.010  (-0.032 – 0.012) | F1-F2 vs. F0 | 0.0053  (-0.018 – 0.029) |
| NTCP | 2 | NASH vs. NDL | -0.0060  (-0.029 to 0.016) | S2 vs. S0 | -0.0052  (-0.031 – 0.018) | I2 vs. I0 | -0.011  (-0.043 – 0.020) | B2 vs. B0 | 0.010  (-0.021 – 0.041) |  | |
| NTCP | 2 |  | | S3 vs. S0 | -0.0050  (-0.035 – 0.026) |  | |  | |  |  |
| NTCP | 3 | NAFL vs. NDL | 0.015  (-0.018 – 0.050) | S1 vs. S0 | 0.0072  (-0.028 – 0.041) | I1 vs. I0 | 0.0095  (-0.016 – 0.036) | B1 vs. B0 | 0.0062  (-0.024 – 0.037) | F1-F2 vs. F0 | 0.0079  (-0.024 – 0.041) |
| NTCP | 3 | NASH vs. NDL | 0.014  (-0.015 – 0.043) | S2 vs. S0 | 0.020  (-0.013 – 0.054) | I2 vs. I0 | 0.045*  (0.0021 – 0.086) | B2 vs. B0 | -0.0032  (-0.045 – 0.040) |  | |
| NTCP | 3 |  | | S3 vs. S0 | 0.022  (-0.020 – 0.065) |  | |  | |  |  |
| OATP1B1 | 1 | NAFL vs. NDL | 0.016  (-0.017 – 0.048) | S1 vs. S0 | 0.0097  (-0.024 – 0.042) | I1 vs. I0 | 0.015  (-0.012 – 0.043) | B1 vs. B0 | 0.0044  (-0.024 – 0.033) | F1-F2 vs. F0 | -0.0095  (-0.039 – 0.022) |
| OATP1B1 | 1 | NASH vs. NDL | 0.0064  (-0.022 – 0.034) | S2 vs. S0 | 0.016  (-0.017 – 0.049) | I2 vs. I0 | -0.015  (-0.057 – 0.026) | B2 vs. B0 | -0.0081  (-0.047 – 0.032) |  | |
| OATP1B1 | 1 |  | | S3 vs. S0 | -0.0026  (-0.041 – 0.039) |  | |  | |  |  |
| OATP1B1 | 2 | NAFL vs. NDL | 0.0153  (-0.022 – 0.053) | S1 vs. S0 | 0.00031  (-0.037 – 0.039) | I1 vs. I0 | 0.0065  (-0.026 – 0.037) | B1 vs. B0 | -0.012  (-0.045 – 0.021) | F1-F2 vs. F0 | -0.0059  (-0.041 – 0.030) |
| OATP1B1 | 2 | NASH vs. NDL | -0.0026  (-0.035 – 0.03) | S2 vs. S0 | 0.0070  (-0.031 – 0.044) | I2 vs. I0 | -0.0071  (-0.057 – 0.041) | B2 vs. B0 | -0.0077  (-0.053 – 0.038 |  | |
| OATP1B1 | 2 |  | | S3 vs. S0 | 0.0071  (-0.041 – 0.054) |  | |  | |  |  |
| OATP1B1 | 3 | NAFL vs. NDL | -0.031  (-0.086 – 0.020) | S1 vs. S0 | -0.010  (-0.064 – 0.049) | I1 vs. I0 | -0.021  (-0.067 – 0.024) | B1 vs. B0 | 0.0070  (-0.042 – 0.055) | F1-F2 vs. F0 | 0.015  (-0.038 – 0.066) |
| OATP1B1 | 3 | NASH vs. NDL | -0.0037  (-0.051 – 0.044) | S2 vs. S0 | -0.023  (-0.080 – 0.032) | I2 vs. I0 | -0.022  (-0.050 – 0.092) | B2 vs. B0 | 0.015  (-0.056 – 0.082) |  | |
| OATP1B1 | 3 |  | | S3 vs. S0 | -0.0050  (-0.074 – 0.065) |  | |  | |  |  |
| OATP1B3 | 1 | NAFL vs. NDL | 0.046*  (0.0024 – 0.10) | S1 vs. S0 | 0.033  (-0.0064 – 0.085) | I1 vs. I0 | 0.039*  (0.0025 – 0.083) | B1 vs. B0 | 0.023  (-0.012 – 0.068) | F1-F2 vs. F0 | 0.014  (-0.022 – 0.063) |
| OATP1B3 | 1 | NASH vs. NDL | 0.039*  (0.0034 – 0.087) | S2 vs. S0 | 0.037  (-0.0020 – 0.089) | I2 vs. I0 | 0.052  (-0.0028 – 0.13) | B2 vs. B0 | 0.0123  (-0.029 – 0.067) |  | |
| OATP1B3 | 1 |  | | S3 vs. S0 | 0.045  (-0.0021 – 0.11) |  | |  | |  |  |
| OATP1B3 | 2 | NAFL vs. NDL | -0.0032  (-0.12 – 0.11) | S1 vs. S0 | -0.023  (-0.14 – 0.083) | I1 vs. I0 | -0.012  (-0.11 – 0.072) | B1 vs. B0 | 0.0097  (-0.089 – 0.11) | F1-F2 vs. F0 | -0.020  (-0.13 – 0.084) |
| OATP1B3 | 2 | NASH vs. NDL | -0.0067  (-0.10 – 0.090) | S2 vs. S0 | -0.0024  (-0.11 – 0.11) | I2 vs. I0 | 0.025  (-0.012 – 0.16) | B2 vs. B0 | -0.030  (-0.16 to 0.10) |  | |
| OATP1B3 | 2 |  | | S3 vs. S0 | 0.015  (-0.11 – 0.15) |  | |  | |  |  |
| OATP1B3 | 3 | NAFL vs. NDL | -0.033  (-0.14 – 0.070) | S1 vs. S0 | -0.012  (-0.14 – 0.11) | I1 vs. I0 | -0.028  (-0.13 – 0.069) | B1 vs. B0 | -0.034  (-0.15 – 0.077) | F1-F2 vs. F0 | 0.0042  (-0.12 – 0.12) |
| OATP1B3 | 3 | NASH vs. NDL | -0.046  (-0.17 – 0.074) | S2 vs. S0 | -0.037  (-0.16 – 0.083 | I2 vs. I0 | -0.081  (-0.24 – 0.077) | B2 vs. B0 | 0.015  (-0.14 – 0.16) |  | |
| OATP1B3 | 3 |  | | S3 vs. S0 | -0.068  (-0.22 – 0.077) |  | |  | |  |  |

*CI, credible interval*

*95% credible interval does not cross 0

**Table S5.** Estimated mean differences (median point estimates and 95% credible intervals) for zonal and overall plasma membrane localization Beta family Bayesian models.

|  | | *NAFLD Diagnosis* | | *Steatosis* | | *Lobular Inflammation* | | *Hepatocellular Ballooning* | | *Fibrosis* | |
| --- | --- | --- | --- | --- | --- | --- | --- | --- | --- | --- | --- |
| **Transporter** | **Zone** | **Comp** | **Median (95% CI)** | **Comp** | **Median (95% CI)** | **Comp** | **Median (95% CI)** | **Comp** | **Median (95% CI)** | **Comp** | **Median (95% CI)** |
| BSEP | 1 | NAFL vs. NDL | 6.36  (-1.63 – 16.90) | S1 vs. S0 | 0.67  (-11.00 – 11.20) | I1 vs. I0 | 2.22  (-7.05 – 11.30) | B1 vs. B0 | -3.51  (-13.00 – 5.62) | F1-F2 vs. F0 | 1.45  (-8.62 – 11.7) |
| BSEP | 1 | NASH vs. NDL | 0.49  (-8.56 – 9.23) | S2 vs. S0 | 3.22  (-6.46 – 13.70) | I2 vs. I0 | 4.74  (-7.95 – 18.70) | B2 vs. B0 | 2.98  (-8.04 – 13.90) |  | |
| BSEP | 1 |  | | S3 vs. S0 | 4.27  (-7.33 – 15.90) |  | |  | |  |  |
| BSEP | 2 | NAFL vs. NDL | 3.32  (-4.59 – 12.80) | S1 vs. S0 | -4.85  (-17.20 – 6.00) | I1 vs. I0 | -2.04  (-11.20 – 7.09) | B1 vs. B0 | -8.00  (-19.10 – 0.85) | F1-F2 vs. F0 | -2.12  (-13.7 – 7.49) |
| BSEP | 2 | NASH vs. NDL | -5.67  (-16.00 – 2.87) | S2 vs. S0 | -0.24  (-10.30 – 9.89) | I2 vs. I0 | -0.96  (-16.00 – 11.30 | B2 vs. B0 | -4.43  (-18.30 – 6.36) |  | |
| BSEP | 2 |  | | S3 vs. S0 | -0.52  (-13.80 – 10.20) |  | |  | |  |  |
| BSEP | 3 | NAFL vs. NDL | -1.40  (-10.70 – 6.74) | S1 vs. S0 | -4.38  (-16.20 – 5.42) | I1 vs. I0 | -5.25  (-15.40 – 2.88) | B1 vs. B0 | -13.30*  (-24.90 to -2.51) | F1-F2 vs. F0 | -0.34  (-11.70 – 9.42) |
| BSEP | 3 | NASH vs. NDL | -8.99*  (-19.80 to -0.66) | S2 vs. S0 | -8.51  (-21.30 – 1.25) | I2 vs. I0 | -9.24  (-26.00 – 3.69) | B2 vs. B0 | 0.16  (-11.5 – 10.30 |  | |
| BSEP | 3 |  | | S3 vs. S0 | -2.84  (-16.60 – 7.21) |  | |  | |  |  |
| NTCP | 1 | NAFL vs. NDL | -0.38  (-6.16 – 5.36) | S1 vs. S0 | -3.59  (-9.02 – 2.03) | I1 vs. I0 | -2.74  (-7.67 – 1.81) | B1 vs. B0 | -2.82  (-7.96 – 1.73) | F1-F2 vs. F0 | -5.07*  (-9.88 to -0.35) |
| NTCP | 1 | NASH vs. NDL | -4.22  (-9.42 – 0.59) | S2 vs. S0 | -0.65  (-5.97 – 5.39) | I2 vs. I0 | -1.27  (-8.67 – 6.00) | B2 vs. B0 | -6.37*  (-12.50 to -0.49) |  | |
| NTCP | 1 |  | | S3 vs. S0 | -3.65  (-10.10 – 2.50) |  | |  | |  |  |
| NTCP | 2 | NAFL vs. NDL | -0.21  (-6.39 – 5.83) | S1 vs. S0 | -2.22  (-8.66 – 3.33) | I1 vs. I0 | -1.65  (-6.76 – 3.28) | B1 vs. B0 | 0.31  (-4.64 – 5.92) | F1-F2 vs. F0 | -2.86  (-7.76 – 2.53) |
| NTCP | 2 | NASH vs. NDL | -1.41  (-6.60 – 3.82) | S2 vs. S0 | 0.90  (-4.78 – 7.19) | I2 vs. I0 | 2.00  (-6.11 – 10.60) | B2 vs. B0 | -3.99  (-10.80 – 2.81 |  | |
| NTCP | 2 |  | | S3 vs. S0 | -2.29  (-8.60 – 4.82) |  | |  | |  |  |
| NTCP | 3 | NAFL vs. NDL | -1.16  (-7.37 – 4.80) | S1 vs. S0 | -2.82  (-9.09 – 3.39) | I1 vs. I0 | -2.79  (-8.01 – 2.34) | B1 vs. B0 | 0.90  (-4.30 – 6.75) | F1-F2 vs. F0 | -5.33*  (-11.10 to -0.56) |
| NTCP | 3 | NASH vs. NDL | -2.63  (-8.16 – 2.45) | S2 vs. S0 | -0.038  (-6.26 – 6.35) | I2 vs. I0 | 1.00  (-7.19 – 9.29) | B2 vs. B0 | -5.25  (-12.30 – 1.50) |  | |
| NTCP | 3 |  | | S3 vs. S0 | -4.20  (-10.80 – 3.02) |  | |  | |  |  |
| OATP1B1 | 1 | NAFL vs. NDL | 6.84  (-3.66 – 19.4) | S1 vs. S0 | -3.87  (-14.2 – 5.88) | I1 vs. I0 | 1.51  (-7.49 – 10.70) | B1 vs. B0 | -1.84  (-12.10 – 8.22) | F1-F2 vs. F0 | -1.02  (-12.80 – 9.60) |
| OATP1B1 | 1 | NASH vs. NDL | 0.44  (-9.07 – 10.1) | S2 vs. S0 | 6.25  (-4.37 – 16.80) | I2 vs. I0 | 12.60  (-3.02 – 29.50) | B2 vs. B0 | -6.31  (-19.80 – 5.88) |  | |
| OATP1B1 | 1 |  | | S3 vs. S0 | 12.20  (-1.62 – 26.5) |  | |  | |  |  |
| OATP1B1 | 2 | NAFL vs. NDL | 5.71  (-5.08 – 17.6) | S1 vs. S0 | -0.73  (-10.80 – 9.48) | I1 vs. I0 | 3.10  (-5.58 – 12.60) | B1 vs. B0 | 0.43  (-9.76 – 10.90) | F1-F2 vs. F0 | 6.68  (-4.67 – 19.50) |
| OATP1B1 | 2 | NASH vs. NDL | 4.53  (-4.39 – 15.4) | S2 vs. S0 | 7.16  (-2.41 – 18.60) | I2 vs. I0 | 10.60  (-4.05 – 27.90) | B2 vs. B0 | 3.07  (-10.30 – 18.20) |  | |
| OATP1B1 | 2 |  | | S3 vs. S0 | 12.10  (-1.32 – 26.60) |  | |  | |  |  |
| OATP1B1 | 3 | NAFL vs. NDL | 4.47  (-5.45 – 16.4) | S1 vs. S0 | -0.49  (-10.60 – 9.00) | I1 vs. I0 | 2.77  (-6.26 – 11.70) | B1 vs. B0 | 1.76  (-6.82 – 13.00) | F1-F2 vs. F0 | 2.88  (-7.43 – 14.70) |
| OATP1B1 | 3 | NASH vs. NDL | 3.79  (-5.38 – 13.7) | S2 vs. S0 | 6.42  (-3.26 – 17.30) | I2 vs. I0 | 7.36  (-6.02 – 24.2) | B2 vs. B0 | 0.39  (-12.50 – 13.60) |  | |
| OATP1B1 | 3 |  | | S3 vs. S0 | 10.10  (-2.58 – 24.20) |  | |  | |  |  |
| OATP1B3 | 1 | NAFL vs. NDL | 7.41  (-4.24 – 19.8) | S1 vs. S0 | 5.97  (-5.94 – 19.10) | I1 vs. I0 | 11.70*  (1.72 – 22.60) | B1 vs. B0 | 5.90  (-3.43 – 16.40) | F1-F2 vs. F0 | 6.58  (-3.76 – 17.20) |
| OATP1B3 | 1 | NASH vs. NDL | 12.00*  (2.12 – 23.6) | S2 vs. S0 | 12.60*  (0.83 – 24.20) | I2 vs. I0 | 5.19  (-10.00 – 20.10) | B2 vs. B0 | 8.16  (-5.13 – 20.7) |  | |
| OATP1B3 | 1 |  | | S3 vs. S0 | 8.82  (-3.42 – 22.40) |  | |  | |  |  |
| OATP1B3 | 2 | NAFL vs. NDL | 4.12  (-6.65 – 15.9) | S1 vs. S0 | 4.88  (-5.63 – 17.70) | I1 vs. I0 | 7.77  (-1.09 – 18.30) | B1 vs. B0 | 4.11  (-4.33 – 14.5) | F1-F2 vs. F0 | 6.66  (-3.59 – 16.50) |
| OATP1B3 | 2 | NASH vs. NDL | 8.46  (-0.89 – 19.2) | S2 vs. S0 | 7.81  (-3.07 – 19.10) | I2 vs. I0 | 3.66  (-11.00 – 17.90) | B2 vs. B0 | 5.52  (-7.46 – 17.20) |  | |
| OATP1B3 | 2 |  | | S3 vs. S0 | 6.83  (-4.05 – 20.50) |  | |  | |  |  |
| OATP1B3 | 3 | NAFL vs. NDL | 0.61  (-12.7 -12.9) | S1 vs. S0 | 2.77  (-10.00 – 15.50) | I1 vs. I0 | 5.44  (-4.22 – 16.40) | B1 vs. B0 | 6.07  (-4.17 – 16.50) | F1-F2 vs. F0 | 5.91  (-5.38 – 17.20) |
| OATP1B3 | 3 | NASH vs. NDL | 6.89  (-3.44 – 17.9) | S2 vs. S0 | 3.86  (-7.95 – 16.40) | I2 vs. I0 | 0.80  (-15.40 – 16.20) | B2 vs. B0 | 7.40  (-6.49 – 21.70) |  | |
| OATP1B3 | 3 |  | | S3 vs. S0 | 6.56  (-6.49 – 20.60) |  | |  | |  |  |
| BSEP | OA | NAFL vs. NDL | 3.14  (-5.25 – 11.90) | S1 vs. S0 | -1.87  (-12.10 – 8.07) | I1 vs. I0 | -0.73  (-9.19 – 7.58) | B1 vs. B0 | -6.60  (-17.30 – 1.25) | F1-F2 vs. F0 | 1.18  (-8.01 – 10.30) |
| BSEP | OA | NASH vs. NDL | -2.9  (-11.90 – 5.51) | S2 vs. S0 | -0.67  (-11.00 – 8.56) | I2 vs. I0 | 0.30  (-12.50 – 12.60) | B2 vs. B0 | 1.48  (-8.79 – 11.40) |  | |
| BSEP | OA |  | | S3 vs. S0 | 1.92  (-8.96 – 12.90) |  | |  | |  |  |
| NTCP | OA | NAFL vs. NDL | 0.32  (-5.80 – 6.06) | S1 vs. S0 | -0.98  (-6.88 – 5.08) | I1 vs. I0 | -1.14  (-6.03 – 4.36) | B1 vs. B0 | 0.37  (-5.06 – 5.67) | F1-F2 vs. F0 | -2.81  (-8.22 – 2.47) |
| NTCP | OA | NASH vs. NDL | -1.12  (-6.50 – 4.02) | S2 vs. S0 | 0.94  (-5.29 – 6.96) | I2 vs. I0 | 1.94  (-5.64 – 10.80) | B2 vs. B0 | -3.45  (-9.85 – 3.42) |  | |
| NTCP | OA |  | | S3 vs. S0 | -2.49  (-9.17 – 4.42) |  | |  | |  |  |
| OATP1B1 | OA | NAFL vs. NDL | 5.02  (-3.93 – 16.10) | S1 vs. S0 | -1.23  (-9.36 – 7.37) | I1 vs. I0 | 2.47  (-4.77 – 10.50) | B1 vs. B0 | 1.03  (-7.68 – 10.00) | F1-F2 vs. F0 | 1.30  (-7.72 – 11.0) |
| OATP1B1 | OA | NASH vs. NDL | 2.91  (-4.48 – 11.90) | S2 vs. S0 | 5.90  (-2.99 – 15.30) | I2 vs. I0 | 9.43  (-3.08 – 23.20) | B2 vs. B0 | -2.21  (-13.50 – 8.99) |  | |
| OATP1B1 | OA |  | | S3 vs. S0 | 10.20  (-1.49 – 22.30) |  | |  | |  |  |
| OATP1B3 | OA | NAFL vs. NDL | 2.58  (-4.68 – 10.00) | S1 vs. S0 | 2.01  (-5.72 – 9.65) | I1 vs. I0 | 4.96  (-1.24 – 11.90) | B1 vs. B0 | 5.01  (-1.39 – 12.20) | F1-F2 vs. F0 | 3.03  (-4.51 – 10.30) |
| OATP1B3 | OA | NASH vs. NDL | 5.88  (-0.62 – 12.6) | S2 vs. S0 | 7.02*  (0.46 – 14.90) | I2 vs. I0 | 2.75  (-7.70 – 12.60) | B2 vs. B0 | 4.70  (-4.30 – 13.10) |  | |
| OATP1B3 | OA |  | | S3 vs. S0 | 4.71  (-4.52 – 12.90) |  | |  | |  |  |

*CI, credible interval; OA, overall*

*95% credible interval does not cross 0. All values are in percentage points.

**Table S6.** Estimated mean differences (median point estimates and 95% credible intervals) for membrane-localized zonal abundance (MZA) Dirichlet family Bayesian models.

|  | | *NAFLD Diagnosis* | | *Steatosis* | | *Lobular Inflammation* | | *Hepatocellular Ballooning* | | *Fibrosis* | |
| --- | --- | --- | --- | --- | --- | --- | --- | --- | --- | --- | --- |
| **Transporter** | **Zone** | **Comp** | **Median (95% CI)** | **Comp** | **Median (95% CI)** | **Comp** | **Median (95% CI)** | **Comp** | **Median (95% CI)** | **Comp** | **Median (95% CI)** |
| BSEP | 1 | NAFL vs. NDL | 0.040  (-0.00021 – 0.080) | S1 vs. S0 | 0.094*  (0.053 – 0.14) | I1 vs. I0 | 0.073*  (0.037 – 0.11) | B1 vs. B0 | 0.075*  (0.039 – 0.11) | F1-F2 vs. F0 | 0.052*  (0.0055 – 0.097) |
| BSEP | 1 | NASH vs. NDL | 0.090*  (0.054 – 0.13) | S2 vs. S0 | 0.040  (-0.0014 – 0.081) | I2 vs. I0 | 0.066*  (0.010 – 0.13) | B2 vs. B0 | 0.089*  (0.040 – 0.14) |  | |
| BSEP | 1 |  | | S3 vs. S0 | 0.077*  (0.024 – 0.13) |  | |  | |  |  |
| BSEP | 2 | NAFL vs. NDL | 0.0021  (-0.042 – 0.047) | S1 vs. S0 | -0.025  (-0.070 – 0.020) | I1 vs. I0 | -0.016  (-0.057 – 0.024) | B1 vs. B0 | -0.012  (-0.051 – 0.025) | F1-F2 vs. F0 | -0.031  (-0.078 – 0.014) |
| BSEP | 2 | NASH vs. NDL | -0.024  (-0.065 – 0.016) | S2 vs. S0 | 0.0065  (-0.038 – 0.049) | I2 vs. I0 | -0.011  (-0.074 – 0.053) | B2 vs. B0 | -0.055*  (-0.10 to -0.0047) |  | |
| BSEP | 2 |  | | S3 vs. S0 | -0.034  (-0.084 – 0.017) |  | |  | |  |  |
| BSEP | 3 | NAFL vs. NDL | -0.042  (-0.096 – 0.011) | S1 vs. S0 | -0.069*  (-0.13 to -0.016) | I1 vs. I0 | -0.056*  (-0.11 to -0.011) | B1 vs. B0 | -0.062*  (-0.11 to -0.017) | F1-F2 vs. F0 | -0.021  (-0.083 – 0.038) |
| BSEP | 3 | NASH vs. NDL | -0.065*  (-0.12 to -0.019) | S2 vs. S0 | -0.046  (-0.10 – 0.0075) | I2 vs. I0 | -0.055  (-0.13 – 0.019) | B2 vs. B0 | -0.035  (-0.099 – 0.029) |  | |
| BSEP | 3 |  | | S3 vs. S0 | -0.045  (-0.11 – 0.023) |  | |  | |  |  |
| NTCP | 1 | NAFL vs. NDL | -0.013  (-0.054 – 0.027) | S1 vs. S0 | -0.023  (-0.065 – 0.18) | I1 vs. I0 | -0.020  (-0.054 – 0.014) | B1 vs. B0 | -0.033  (-0.067 – 0.0020) | F1-F2 vs. F0 | -0.029  (-0.066 – 0.0085) |
| NTCP | 1 | NASH vs. NDL | -0.037*  (-0.073 to -0.0016) | S2 vs. S0 | -0.031  (0.071 – 0.010) | I2 vs. I0 | -0.067*  (-0.12 to -0.018) | B2 vs. B0 | -0.033  (-0.080 – 0.016) |  | |
| NTCP | 1 |  | | S3 vs. S0 | -0.032  (-0.080 – 0.018) |  | |  | |  |  |
| NTCP | 2 | NAFL vs. NDL | -0.0043  (-0.046 – 0.039) | S1 vs. S0 | 0.013  (-0.032 – 0.057) | I1 vs. I0 | 0.00332  (-0.033 – 0.039) | B1 vs. B0 | -0.0043  (-0.040 – 0.033) | F1-F2 vs. F0 | 0.034  (-0.010 – 0.075) |
| NTCP | 2 | NASH vs. NDL | 0.0075  (-0.030 – 0.047) | S2 vs. S0 | -0.0027  (-0.046 – 0.040) | I2 vs. I0 | -0.0020  (-0.058 – 0.057) | B2 vs. B0 | 0.043  (-0.011 – 0.093) |  | |
| NTCP | 2 |  | | S3 vs. S0 | -0.0080  (-0.061 – 0.044) |  | |  | |  |  |
| NTCP | 3 | NAFL vs. NDL | 0.016  (-0.042 – 0.073) | S1 vs. S0 | 0.010  (-0.047 – 0.069) | I1 vs. I0 | 0.017  (-0.028 – 0.064) | B1 vs. B0 | 0.037  (-0.014 – 0.085) | F1-F2 vs. F0 | -0.0051  (-0.059 – 0.050) |
| NTCP | 3 | NASH vs. NDL | 0.030  (-0.021 – 0.080) | S2 vs. S0 | 0.034  (-0.026 – 0.092) | I2 vs. I0 | 0.068  (-0.0060 – 0.14) | B2 vs. B0 | -0.011  (-0.077 – 0.056) |  | |
| NTCP | 3 |  | | S3 vs. S0 | 0.040  (-0.030 – 0.11) |  | |  | |  |  |
| OATP1B1 | 1 | NAFL vs. NDL | 0.046*  (0.0015 – 0.092) | S1 vs. S0 | -0.022  (-0.066 – 0.022) | I1 vs. I0 | 0.0085  (-0.030 – 0.048) | B1 vs. B0 | -0.043*  (-0.081 to -0.0042) | F1-F2 vs. F0 | -0.031  (-0.073 – 0.012) |
| OATP1B1 | 1 | NASH vs. NDL | -0.018  (-0.055 – 0.020) | S2 vs. S0 | 0.027  (-0.020 – 0.073) | I2 vs. I0 | -0.013  (-0.073 – 0.047) | B2 vs. B0 | -0.055*  (-0.11 to -0.0035) |  | |
| OATP1B1 | 1 |  | | S3 vs. S0 | 0.024  (-0.032 – 0.080) |  | |  | |  |  |
| OATP1B1 | 2 | NAFL vs. NDL | 0.0043  (-0.049 – 0.054) | S1 vs. S0 | 0.010  (-0.046 – 0.065) | I1 vs. I0 | 0.012  (-0.032 – 0.058) | B1 vs. B0 | -0.015  (-0.059 – 0.031) | F1-F2 vs. F0 | 0.044  (-0.0084 – 0.099) |
| OATP1B1 | 2 | NASH vs. NDL | 0.012  (-0.034 – 0.058) | S2 vs. S0 | 0.001  (-0.050 – 0.055) | I2 vs. I0 | -0.0076  (-0.078 – 0.065) | B2 vs. B0 | 0.049  (-0.020 – 0.12) |  | |
| OATP1B1 | 2 |  | | S3 vs. S0 | 0.020  (-0.041 – 0.087) |  | |  | |  |  |
| OATP1B1 | 3 | NAFL vs. NDL | -0.052  (-0.12 – 0.023) | S1 vs. S0 | 0.012  (-0.066 – 0.091) | I1 vs. I0 | -0.021  (-0.085 – 0.046) | B1 vs. B0 | 0.058  (-0.011 – 0.12) | F1-F2 vs. F0 | -0.015  (-0.087 – 0.063) |
| OATP1B1 | 3 | NASH vs. NDL | 0.0059  (-0.061 – 0.071) | S2 vs. S0 | -0.028  (-0.10 – 0.051) | I2 vs. I0 | 0.020  (-0.085 – 0.12) | B2 vs. B0 | 0.0054  (-0.082 – 0.096) |  | |
| OATP1B1 | 3 |  | | S3 vs. S0 | -0.046  (-0.13 – 0.044) |  | |  | |  |  |
| OATP1B3 | 1 | NAFL vs. NDL | 0.041*  (0.0024 – 0.0960 | S1 vs. S0 | 0.033  (-0.0033 – 0.083) | I1 vs. I0 | 0.043*  (0.00742 – 0.091) | B1 vs. B0 | 0.029  (-0.0064 – 0.081) | F1-F2 vs. F0 | 0.23  (-0.017 – 0.075) |
| OATP1B3 | 1 | NASH vs. NDL | 0.047*  (0.010 – 0.095) | S2 vs. S0 | 0.043*  (0.0021 – 0.096) | I2 vs. I0 | 0.050  (-0.00297 – 0.12) | B2 vs. B0 | 0.019  (-0.028 – 0.080) |  | |
| OATP1B3 | 1 |  | | S3 vs. S0 | 0.050  (-0.0011 – 0.12) |  | |  | |  |  |
| OATP1B3 | 2 | NAFL vs. NDL | 0.0096  (-0.11 – 0.12) | S1 vs. S0 | -0.0038  (-0.12 – 0.11) | I1 vs. I0 | 0.0022  (-0.094 – 0.097) | B1 vs. B0 | 0.0030  (-0.099 – 0.11) | F1-F2 vs. F0 | -0.0053  (-0.12 – 0.10) |
| OATP1B3 | 2 | NASH vs. NDL | 0.0063  (-0.095 – 0.11) | S2 vs. S0 | -0.000062  (-0.12 – 0.11) | I2 vs. I0 | 0.029  (-0.12 – 0.17) | B2 vs. B0 | -0.032  (-0.17 – 0.11) |  | |
| OATP1B3 | 2 |  | | S3 vs. S0 | 0.029  (-0.11 – 0.17) |  | |  | |  |  |
| OATP1B3 | 3 | NAFL vs. NDL | -0.053  (-0.18 – 0.069) | S1 vs. S0 | -0.032  (-0.16 – 0.090) | I1 vs. I0 | -0.047  (-0.15 – 0.058) | B1 vs. B0 | -0.036  (-0.15 – 0.077) | F1-F2 vs. F0 | -0.020  (-0.14 – 0.11) |
| OATP1B3 | 3 | NASH vs. NDL | -0.055  (-0.17 – 0.054) | S2 vs. S0 | -0.044  (-0.17 – 0.082) | I2 vs. I0 | -0.083  (-0.25 – 0.079) | B2 vs. B0 | 0.011  (-0.15 – 0.16) |  | |
| OATP1B3 | 3 |  | | S3 vs. S0 | -0.083  (-0.24 – 0.062) |  | |  | |  |  |

**References**

[1.    Nies AT, Schaeffeler E, Schwab M. Hepatic solute carrier transporters and drug therapy: Regulation of expression and impact of genetic variation. *Pharmacol Ther*. 2022;238:108268.](https://sciwheel.com/work/bibliography/15461983)

[2.    Croce AC, Ferrigno A, Vairetti M, Bertone R, Freitas I, Bottiroli G. Autofluorescence properties of isolated rat hepatocytes under different metabolic conditions. *Photochem Photobiol Sci*. 2004;3(10):920-926.](https://sciwheel.com/work/bibliography/13390789)

[3.    Croce AC, Ferrigno A, Bottiroli G, Vairetti M. Autofluorescence-based optical biopsy: An effective diagnostic tool in hepatology. *Liver Int*. 2018;38(7):1160-1174.](https://sciwheel.com/work/bibliography/13391590)

[4.    Saran C, Fu D, Ho H, et al. A novel differentiated HuH-7 cell model to examine bile acid metabolism, transport and cholestatic hepatotoxicity. *Sci Rep*. 2022;12(1):14333.](https://sciwheel.com/work/bibliography/13534038)

[5.    Powell J, Farasyn T, Köck K, et al. Novel mechanism of impaired function of organic anion-transporting polypeptide 1B3 in human hepatocytes: post-translational regulation of OATP1B3 by protein kinase C activation. *Drug Metab Dispos*. 2014;42(11):1964-1970.](https://sciwheel.com/work/bibliography/4907)

[6.    Crowe A, Zheng W, Miller J, et al. Characterization of plasma membrane localization and phosphorylation status of organic anion transporting polypeptide (OATP) 1B1 c.521 T>C nonsynonymous single-nucleotide polymorphism. *Pharm Res*. 2019;36(7):101.](https://sciwheel.com/work/bibliography/11156609)

[7.    Arzt M, Deschamps J, Schmied C, et al. LABKIT: labeling and segmentation toolkit for big image data. *Front Comput Sci*. 2022;4:777728.](https://sciwheel.com/work/bibliography/12654452)

[8.    Bürkner P-C. brms: an*R* package for bayesian multilevel models using *stan*. *J Stat Softw*. 2017;80(1):1-28.](https://sciwheel.com/work/bibliography/4604908)

[9.    Segovia-Miranda F, Morales-Navarrete H, Kücken M, et al. Three-dimensional spatially resolved geometrical and functional models of human liver tissue reveal new aspects of NAFLD progression. *Nat Med*. 2019;25(12):1885-1893.](https://sciwheel.com/work/bibliography/8201513)

[10.   Hall A, Covelli C, Manuguerra R, et al. Transaminase abnormalities and adaptations of the liver lobule manifest at specific cut-offs of steatosis. *Sci Rep*. 2017;7:40977.](https://sciwheel.com/work/bibliography/5118494)
